# Sirt6 attenuates chondrocyte senescence and osteoarthritis progression

**DOI:** 10.1038/s41467-022-35424-w

**Published:** 2022-12-10

**Authors:** Ming-liang Ji, Hua Jiang, Zhuang Li, Rui Geng, Jun Zheng Hu, Yu Cheng Lin, Jun Lu

**Affiliations:** 1 The Center of Joint and Sports Medicine, Orthopedics Department, Zhongda Hospital, Southeast University, Nanjing, China; 2 Department of Spine Surgery, The First Affiliated Hospital of Guangxi Medical University, Nanning, China

**Keywords:** Cartilage, Mechanisms of disease, Senescence

## Abstract

Sirt6 has been implicated as a key regulator in aging-related diseases, including osteoarthritis. However, its functional role and molecular mechanism in chondrocyte senescence and osteoarthritis pathophysiology remain largely undefined. Here we show that Sirt6 deficiency exaggerates chondrocyte senescence and osteoarthritis progression, whereas intra-articular injection of adenovirus-Sirt6 markedly attenuates surgical destabilization of medial meniscus-induced osteoarthritis. Mechanistically, Sirt6 can directly interact with STAT5 and deacetylate STAT5, thus inhibiting the IL-15/JAK3-induced STAT5 translocation from cytoplasm to nucleus, which inactivates IL-15/JAK3/STAT5 signaling. Mass spectrometry revealed that Sirt6 deacetylated conserved lysine 163 on STAT5. Mutation of lysine 163 to arginine in STAT5 abolished the regulatory effect of Sirt6. In vivo, specific ablation of Sirt6 in chondrocytes exacerbated osteoarthritis. Pharmacological activation of Sirt6 substantially alleviated chondrocyte senescence. Taken together, Sirt6 attenuates chondrocyte senescence by inhibiting IL-15/JAK3/STAT5 signaling. Targeting Sirt6 represents a promising new approach for osteoarthritis.

## Abstract (web summary)

Ji and colleagues identify Sirt6 as a regulator of chondrocyte senescence. Mechanistically, Sirt6 physically interacts with STAT5 and deacetylates it at K163, which reduces the IL-15/JAK3-induced STAT5 translocation from cytoplasm to nucleus.

## Introduction

Osteoarthritis (OA), the most common form of arthritis, is primarily characterized by cartilage degradation, synovial inflammation, and subchondral bone remodeling, ultimately causing pain and functional disability^1–3^. It is estimated that the number of people affected will be projected to double by 2030^4–6^. However, no disease-modifying osteoarthritis drugs (DMOADs) are currently available^7,8^, which is ascribed to our lack of enhanced understanding of the pathogenesis of OA. Cellular senescence, a permanent state of cell-cycle arrest induced by cellular stress, has recently emerged as a fundamental mechanism that substantially contributes to OA phenotype^9^. Most likely, the thread tying senescence and OA pathology together is the accumulation of senescent chondrocytes over time combined with gradual changes in cellular metabolism, morphology, and function, all of which cause loss of cartilage homeostasis and integrity^10–12^. Therefore, elucidating molecular mechanisms underpinning chondrocyte senescence could lead to the discovery of novel therapeutic strategies for slowing or stopping the progression of OA.

Sirt6, a member of the Sirtuin family of NAD+ -dependent enzymes, shares conserved core catalytic domains, but differs in their cellular localization and tissue distribution, which has been implicated in aging and senescence-associated diseases^13–15^. Wu et al.^16^ found that overexpression of Sirt6 can prevent OA development by reducing both the inflammatory response and chondrocytes senescence. Further, Nagai et al.^17^ revealed that depletion of Sirt6 in human chondrocytes caused increased DNA damage and telomere dysfunction, and subsequent premature senescence. Notably, Sirt6-deficient mice have shortened lifespans and phenotypes associated with aging, cancer, and metabolic disorders^18,19^. Lee et al.^20^ reported that mice treated with Ad-Sirt6 exhibited attenuated severity of arthritis based on clinical scores, hind paw thickness, and radiographic and pathologic findings. Moreover, the injection of Ad-SIRT6 can significantly decrease local and systemic levels of proinflammatory cytokines via blocking the NF-_K_B signaling pathway. However, to the best of our knowledge, the precise molecular mechanisms of Sirt6 in chondrocyte senescence and OA progression remain unclear.

This study explored the essential role and underlying molecular mechanism of Sirt6 in chondrocyte senescence and OA progression. Using clinical samples from patients with OA and controls, we identified that Sirt6 was significantly decreased in cartilage tissues and chondrocytes. We extensively investigated the effect of Sirt6 on chondrocyte senescence both in vivo and in vitro. We verified that Sirt6 prevented OA in our models, and elaborated on the molecular mechanism by which it does so. These findings highlight the critical role of Sirt6 in chondrocyte senescence and indicate that Sirt6 may act as a novel potential therapeutic target for OA.

## Results

### Sirt6 is an essential factor for chondrocyte senescence and OA pathogenesis

**Fig. 1:**
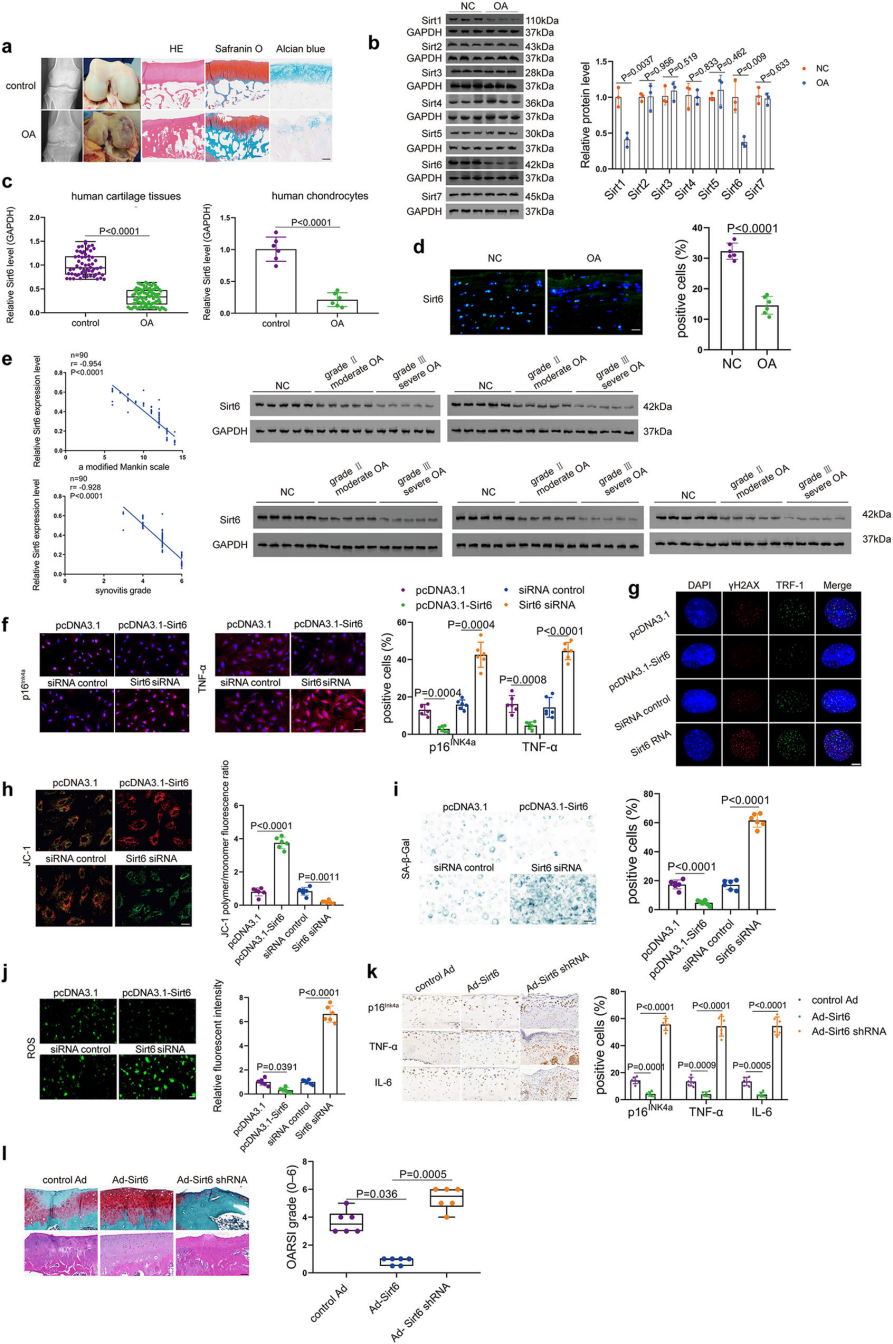
Downregulation of Sirt6 is associated with chondrocyte senescence and OA. **a** Representative radiographic images and gross appearance of the cartilage of OA patients and controls, H&E, Safranin O, and Alcian blue staining. *n* = 6 independent biological replicates per group. **b** Representative images of western blot gel and quantitative analysis. *n* = 3 independent biological replicates per group. **c** Compared with controls (*n* = 60), Sirt6 level was downregulated in cartilage tissues of OA patients (*n* = 90). In chondrocytes of OA patients, decreased level of Sirt6 was also observed (*n* = 6 independent biological replicates per group). **d** Immunofluorescence analysis showing the expression of Sirt6 in the cartilage tissues from OA patients and controls. *n* = 6 independent biological replicates per group. **e** Significant correlations between Sirt6 and a modified Mankin grade and synovitis grade were observed. The association between Sirt6 and OA severity was also validated by western blot (*n* = 25 independent biological replicates per group). **f**–**j** Representative images showing the p16^INK4a^ and TNF-α expression levels (**f**), DNA damage (**g**), mitochondrial membrane potential (**h**), SA-β-Gal positivity (**i**), and ROS level (**j**) in human OA chondrocytes that were transfected by pcDNA3.1-Sirt6, Sirt6 siRNA or their corresponding controls and quantification. *n* = 6 independent biological replicates per group. **k** Immunohistochemistry staining of representative images of indicated markers in articular cartilage of DMM-induced mice treated by Ad-Sirt6, Ad-Sirt6 shRNA, or control Ad. *n* = 6 mice per group. **l** Safranin O/fast green and H&E staining of knee joints of DMM-induced mice receiving different treatments. *n* = 6 mice per group. Scar bar: **a** 200 μm, **l** 50 μm, **d**, **f**, **h**–**k** 20 μm, **g** 5 μm. Data were presented as the mean ± s.e.m (**b**, right panel of **c**, and **f**–**k**) or median (25–75th percentiles) (left panel of **c**, **l**). *P* values are from two-tailed Mann–Whitney *U*-test (left panel of **c**), two-tailed unpaired *t*-test (**b**, right panel of **c**, **d**), or two-tailed Spearman’s correlation test (left panel of **e**), one-way ANOVA test followed by Tukey’s post hoc test (TNF-α level in **f, i** and p16^INK4a^ level in **k**), Brown–Forsythe and Welch ANOVA test followed by Tamhane’s T2 post hoc analysis (**h**, **j**, p16^INK4a^ level in **f**, TNF-α and IL-6 levels in **k**) or Kruskal–Wallis test followed by Dunn’s post hoc test (**l**). Source data are provided as a Source Data file.

We first assessed the expression patterns of SIRT families in cartilage tissues from OA patients and controls (Fig. 1a), and the results demonstrated that Sirt1 and Sirt6 were significantly downregulated (Fig. 1b). Considering that both Sirt1 and Sirt6 were reduced in cartilage tissues of OA patients and recent studies have highlighted the contribution of Sirt1 to the regulation of OA development^21–24^, this study was specially designed to further explore the role of Sirt6 in chondrocyte senescence and OA progression. Interestingly, real-time RT-PCR analysis exhibited a significantly decreased level of Sirt6 in cartilage tissues and chondrocytes from OA patients and controls (Fig. 1c). Moreover, Sirt1, 2, 3, 4, 5, and 7 were also detected by qRT-PCR in cartilage tissues from OA patients and controls (Supplementary Fig. 1a). Immunofluorescence staining further confirmed decreased expression of Sirt6 (Fig. 1d). Correlations between Sirt6 level and the modified Mankin scale and synovitis were observed (Fig. 1e). The western blot analysis also indicated the association between Sirt6 and OA severity (Fig. 1e). Given the critical role of Sirt6 in cellular senescence^13–15^, we explored a possible relationship between Sirt6 and chondrocyte senescence. Overexpression or deletion of Sirt6 remarkably affects p16^INK4a^ and senescence-associated secretory phenotype (SASP) expressions (Fig. 1f and Supplementary Fig. 1b). More importantly, human OA chondrocytes transfected with Sirt6 siRNA had increased DNA damage (Fig. 1g and Supplementary Fig. 1c), decreased mitochondrial membrane potential (Fig. 1h and Supplementary Fig. 1d) and increased senescence-associated-galactosidase (SA-β-Gal) positivity (Fig. 1i and Supplementary Fig. 1e), reactive oxygen species (ROS) level (Fig. 1j and Supplementary Fig. 1f) and shorter telomeres (Supplementary Fig. 1g) than those overexpressing Sirt6. Further, surgical destabilization of the medial meniscus (DMM) was established in wild-type mice, followed by intra-articular injection of control, adeno-associated virus (Ad) Sirt6, or Ad-Sirt6 shRNA once a week for 3 weeks. OA phenotypes were assessed at 8 weeks post-OA surgery. The efficiency of the in vivo Sirt6 knockout and overexpression was analyzed (Supplementary Fig. 1h). Compared with the Ad-Sirt6 treatment group, p16^INK4a^, TNF-α, and IL-6 expressions were markedly elevated in Ad-Sirt6 shRNA group at 8 weeks (Fig. 1k and Supplementary Fig. 1i). Moreover, significantly more severe OA lesions were observed in Ad-Sirt6 shRNA (Fig. 1l and Supplementary Fig. 1j). These findings imply that Sirt6 acts as a crucial regulator in chondrocyte senescence and OA progression.

### Targeted deletion of Sirt6 in chondrocytes impairs cartilage development

**Fig. 2:**
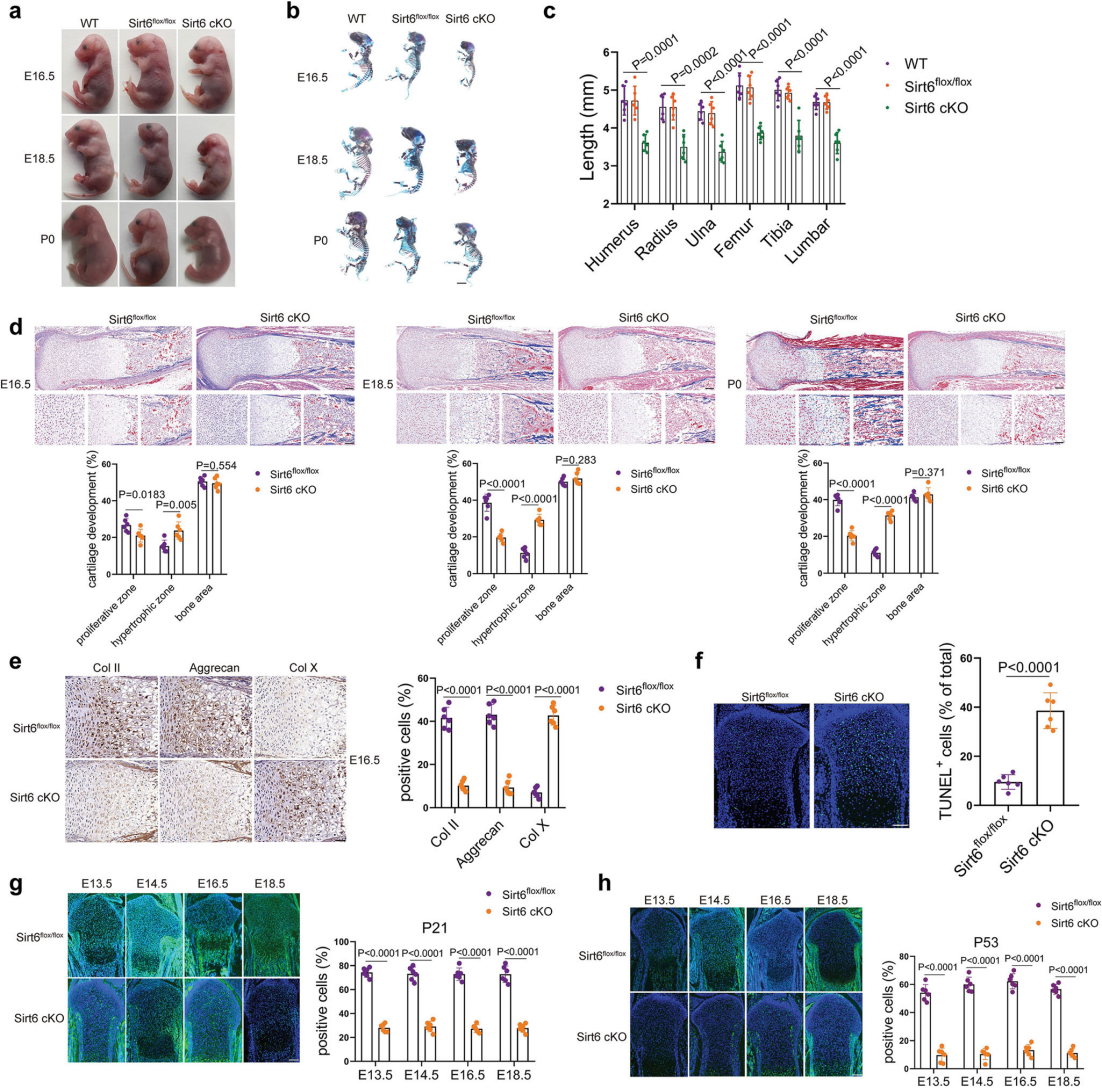
Sirt6 regulates cartilage development and embryonic chondrocyte senescence. **a**–**c** Gross appearance of Sirt6^flox/flox^ and Sirt6 cKO (**a**), double staining with alizarin red and alcian blue of the whole skeleton of Sirt6^flox/flox^ and Sirt6 cKO littermate embryos (E16.5, E18.5, and P0) (**b**) and length of long bones and vertebra (first to fifth lumbar spines) of Sirt6^flox/flox^ and Sirt6 cKO littermate embryos (E18.5) (**c**). *n* = 6 mice per group. **d** Masson trichrome staining of whole tibias. The percentage of the length of the bone area, hypertrophic zone, and proliferative zone over the total tibia length of Sirt6^flox/flox^ and Sirt6 cKO littermate embryos (E16.5, E18.5, and P0). *n* = 6 mice per group. **e** Representative immunohistochemistry of Col II, Aggrecan, and Col X in the tibia of the Sirt6^flox/flox^ and Sirt6 cKO mice and quantitative analysis. *n* = 6 mice per group. **f** TUNEL assays in tibia sections of Sirt6^flox/flox^ and Sirt6 cKO mice at E16.5 and quantitative analysis. *n* = 6 mice per group. **g**, **h** Representative images of immunostaining of P21 and P53 in tibias of Sirt6^flox/flox^ and Sirt6 cKO mice and quantitative analysis (E13.5, E14.5, E16.5, and E18.5). *n* = 6 mice per group. Scar bar: **b** 1 mm, **d** (upper) 200 µm, **f**, **g**, **h** 100 µm, **d** (lower), **e** 50 µm. Data were presented as the mean ± s.e.m. *P* values are from a one-way ANOVA test followed by Tukey’s post hoc test (**c**) or two-tailed unpaired Student’s *t*-test (**d**–**h**). Source data are provided as a Source Data file.

To explore the critical role of the endogenous Sirt6 gene in cartilage development, Col2a1-CreER^T2^ mice with Sirt6^flox/flox^ mice generate cartilage-specific Sirt6 KO mice. Pregnant mice with embryos at E10.5 were injected with tamoxifen (TM). The KO efficiency of Sirt6 was confirmed in Col2a1-CreER^T2^/Sirt6^flox/flox^ (Sirt6 cKO) mice (Supplementary Fig. 2a). Notably, the size of Sirt6 cKO mice skeleton was smaller than that of Sirt6^flox/flox^ embryos (Fig. 2a–c). Moreover, histological examinations were performed in E16.5, E18.5, and P0 embryos. An increased percentage of the hypertrophic zone was observed in the limb of Sirt6 cKO mice (Fig. 2d and Supplementary Fig. 2b). Immunohistochemistry staining also showed weaker positive signals for Col II and Aggrecan, whereas Col X signal was stronger in Sirt6 cKO mice than Sirt6^flox/flox^ mice at E16.5, E18.5, and P0 embryos (Fig. 2e and Supplementary Fig. 2c). In situ TUNEL assays was also performed to detect apoptotic changes in the cartilage growth plate of Sirt6 cKO mice (E16.5) (Fig. 2f). Interestingly, we found that the expression level of P21 and P53, molecular mediators for embryonic senescence^25,26^, was significantly reduced at E13.5, E14.5, E16.5, and E18.5 of Sirt6 cKO mice compared with Sirt6^flox/flox^ (Fig. 2g, h), indicating that Sirt6 could determine the fate of embryonic chondrocyte senescence. Taken together, these data suggest that Sirt6 plays a pivotal role in cartilage development by regulating embryonic chondrocyte senescence.

### Sirt6 regulates aging and injury-induced chondrocyte senescence during OA progression

**Fig. 3:**
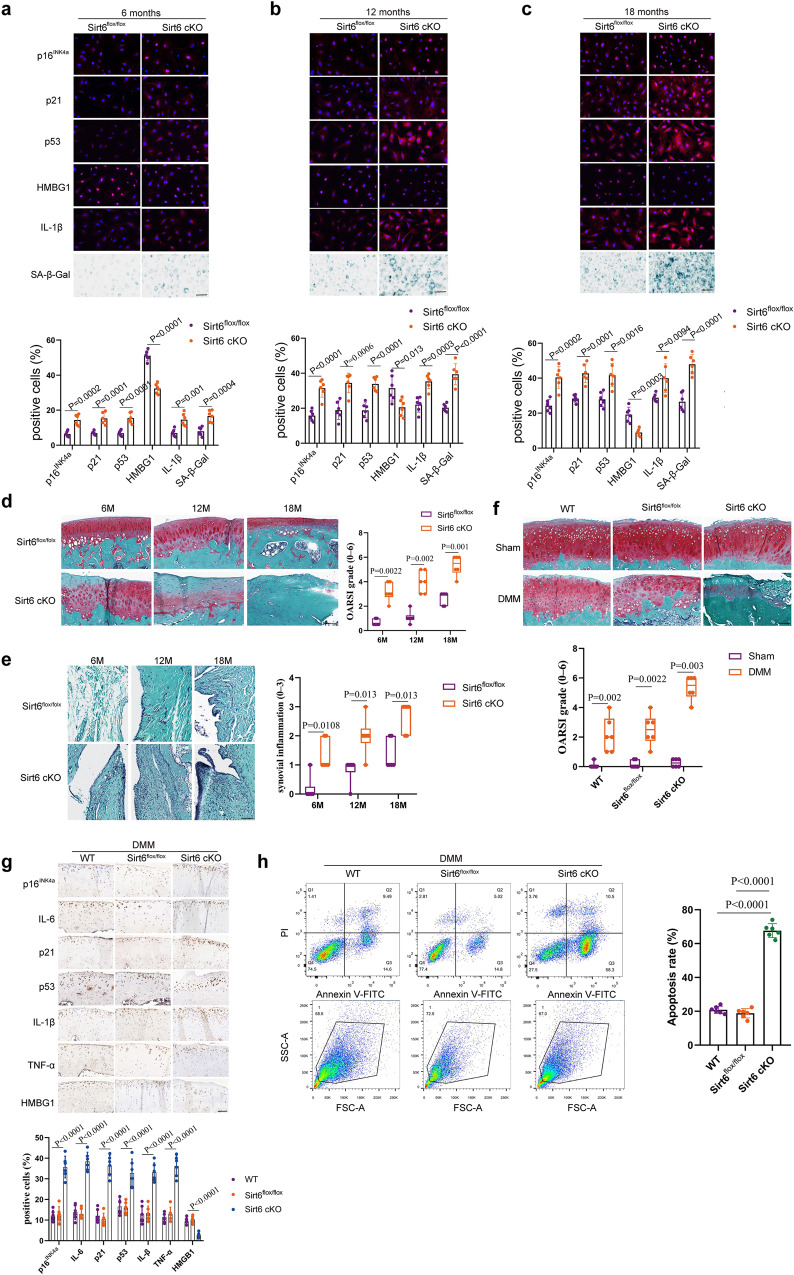
Sirt6 contributes to aging and injury-induced chondrocyte senescence. **a**–**c** Representative images of immunofluorescence of p16^INK4a^, p21, p53, HMBG1, IL-1β, and SA-β-Gal staining in cultured chondrocytes from 6-month-old mice (Sirt6^flox/flox^ and Sirt6 cKO) (**a**), 12-month-old mice (Sirt6^flox/flox^ and Sirt6 cKO) (**b**), and 18-month-old mice cartilage tissues (Sirt6^flox/flox^ and Sirt6 cKO) (**c**), respectively. *n* = 6 mice per group. **d** Representative images of Safranin O staining of cartilage tissues (medial femoral condyle) from 6-month-old mice (Sirt6^flox/flox^ and Sirt6 cKO), 12-month-old mice (Sirt6^flox/flox^ and Sirt6 cKO), and 18-month-old mice (Sirt6^flox/flox^ and Sirt6 cKO) and OARSI. *n* = 6 mice per group. **e** Synovial inflammation of 6-month-old mice (Sirt6^flox/flox^ and Sirt6 cKO), 12-month-old mice (Sirt6^flox/flox^ and Sirt6 cKO), and 18-month-old mice (Sirt6^flox/flox^ and Sirt6 cKO). *n* = 6 mice per group. **f** Representative images of Safranin O/fast green staining in cartilage tissues (medial femoral condyle) from the indicated groups (WT, Sirt6^flox/flox^, and Sirt6 cKO mice undergoing sham or DMM surgery) and OARSI. *n* = 6 mice per group. **g** Representative images of immunohistochemistry of p16^INK4a^, IL-6, p21, p53, IL-β, TNF-α, and HMGB1 in cartilage tissues (medial femoral condyle) from the indicated groups (WT, Sirt6^flox/flox^ and Sirt6 cKO mice subjected to DMM surgery) at 8 weeks post-surgery. *n* = 6 mice per group. **h** Chondrocytes apoptosis was assayed by flow cytometry in the indicated groups (WT, Sirt6^flox/flox^, and Sirt6 cKO mice subjected to DMM surgery). *n* = 6 independent biological replicates per group. Scar bar: **d**–**f** 50 μm, **a**–**c**, **g** 20 μm. Data were presented as the mean ± s.e.m (**a**–**c**, **g**, **h**) or median (25–75th percentiles) (**d**–**f**). *P* values are from two-tailed unpaired Student’s *t*-test (**c**, p16^INK4a^, p53, HMBG1, IL-1β, and SA-β-Gal levels in **a**, p16^INK4a^, p21, p53, HMBG1, and IL-1β levels in **b**), two-tailed unpaired *t*-test with Welch’s correction (p21 level in **a**, SA-β-Gal level in **b**), two-tailed Mann–Whitney *U*-test (**d**–**f**) or one-way ANOVA test followed by Tukey’s post hoc test (**g**, **h**). Source data are provided as a Source Data file.

Given the importance of Sirt6 in embryonic chondrocyte senescence, we speculated that Sirt6 could modulate adult senescent chondrocytes in age-associated and post-traumatic OA development. We observed spontaneously developed OA in Sirt6 cKO mice with aging. Sirt6^flox/flox^ and Sirt6 cKO (8-week-old) mice were injected intraperitoneally with tamoxifen (100 μg/g body weight) daily for 5 days. Of note, remarkable senescence phenotypes and SASP, such as induction of p16^INK4a^, p21, p53, and IL-1β, downregulation of HMBG1 and increased SA-β-Gal positivity, were detected at postnatal month 6 (P6M) (Fig. 3a), P12M (Fig. 3b), and P18M (Fig. 3c) in Sirt6 cKO mice compared with Sirt6^flox/flox^ mice. Furthermore, histological analysis was performed in Sirt6^flox/flox^ and Sirt6 cKO at P6M, P12M, and P18M. Sirt6 cKO mice showed greater loss of proteoglycans, roughening of the articular cartilage, and the loss of articular chondrocyte cellularity compared to Sirt6^flox/flox^ mice, which were confirmed by OARSI and synovitis scores (Fig. 3d, e).

Before surgical induction of OA, wildtype (WT), Sirt6^flox/flox^, and Sirt6 cKO (8-week-old) mice were injected intraperitoneally with tamoxifen (100 μg/g body weight) daily for 5 days. Then, a surgically induced DMM OA model was performed on the knee joints of them (10-week-old). Sirt6 cKO mice presented markedly more severe cartilage destruction and OARSI grades at 8 weeks after DMM surgery (Fig. 3f). Compared with WT and Sirt6^flox/flox^ mice, senescence markers and SASPs (p16^INK4a^, p21, p53, IL-1β, IL-6, and TNF-α) was significantly increased in Sirt6 cKO mice (Fig. 3g), while HMGB1 level was downregulated. Moreover, enhanced chondrocyte apoptosis was detected in Sirt6 cKO mice (Fig. 3h). In the sham group, no significant difference was observed among WT, Sirt6^flox/flox^, and Sirt6 cKO mice in terms of p16^INK4a^, IL-6, TNF-α, HMGB1, and chondrocyte apoptosis (Supplementary Fig. 3a, b). Our results collectively indicate that Sirt6 governs senescent phenotypes of chondrocytes to protect against OA development.

### IL-15/JAK3/STAT5 axis is critically involved in chondrocyte senescence

**Fig. 4:**
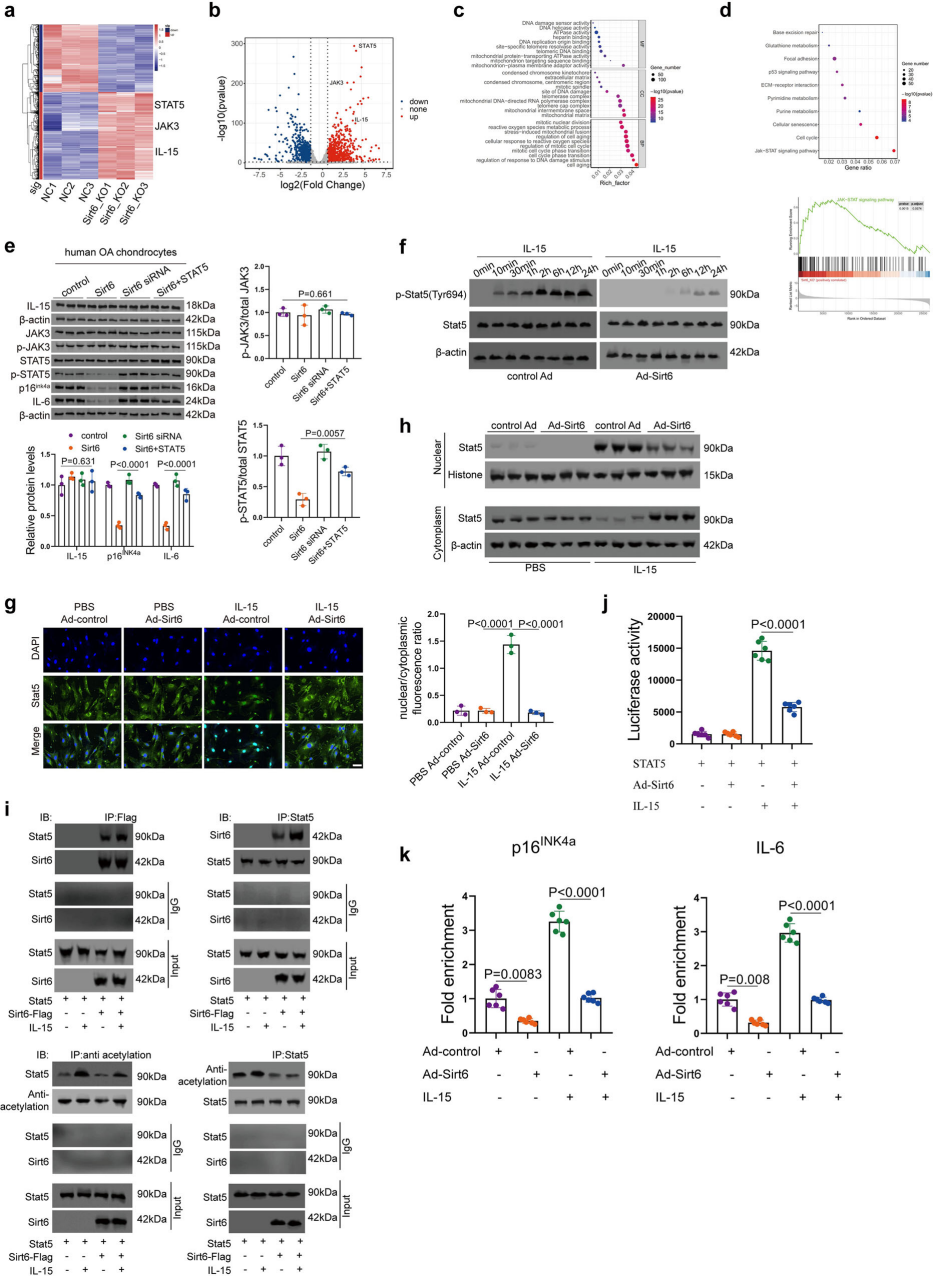
Sirt6 inhibits chondrocyte senescence by regulating the IL-15/JAK3/STAT5 signaling pathway. **a**, **b** Heat map (**a**) and Volcano plot (**b**) demonstrating differentially expressed genes (fold change >2 or <0.5, Benjamini–Hochberg-corrected *p*) in Sirt6 KO chondrocytes vs controls. Notably, STAT5, JAK3, and IL-15 were observed to be significantly upregulated. **c** GO analysis of upregulated genes from Sirt6 KO chondrocytes for biological processes (BP), cellular component (CC), and molecular function (MF). **d** KEGG and GSEA analysis demonstrating JAK/STAT signaling pathway enriched in OA. **e** The rescue experiments were performed in human OA chondrocytes to validate the relationship between Sirt6 and IL-15/JAK3/STAT5 signaling pathway. pcDNA3.1-Sirt6, Sirt6 siRNA, or pcDNA3.1-Sirt6 + pcDNA3.1-STAT5 was transfected into human OA chondrocytes. After 48 h, the related genes were analyzed. *n* = 3 independent biological replicates per group. **f**–**h** Human chondrocytes were infected with Ad-control or Ad-Sirt6, then treated with or without IL-15 (2 ng/mL) for the indicated time. *n* = 3 independent biological replicates per group (**f**). Immunofluorescent staining of cellular Stat5 and nuclear and cytoplasm fraction of Stat5 (**g**). *n* = 3 independent biological replicates per group. Western blot analysis of p-Stat5 and Stat5 expression level. *n* = 3 independent biological replicates per group (**h**). **i** Human chondrocytes were transfected with Stat5, then treated with or without IL-15 (2 ng/mL). Whole-cell lysates were immunoprecipitated with anti-Sirt6 or anti-Stat5 antibodies, and precipitated proteins were detected by anti-Stat5 or anti-Sirt6 antibodies, respectively. Western blot detection of nuclear acetylation level of Stat5 in human chondrocytes with or without IL-15 treatment. *n* = 3 independent biological replicates per group. **j** Human chondrocytes were transfected with the Stat5 reporter and Stat5 with or without Sirt6, then treated with or without IL-15 for 12 h. Luciferase activity was normalized to β-gal. *n* = 6 independent biological replicates per group. **k** ChIP assay analysis of human chondrocytes infected with Ad-control or Ad-Sirt6, then treated with or without IL-15. *n* = 6 independent biological replicates per group. Scar bar: **g** 20 μm. Data were presented as the mean ± s.e.m. *P* values are from two-tailed Mann–Whitney *U*-test (**a**–**d**), one-way ANOVA test followed by Tukey’s post hoc test (**e**, **g**) or Brown–Forsythe and Welch ANOVA test followed by Tamhane’s T2 post hoc analysis (**j**, **k**). Source data are provided as a Source Data file.

To comprehensively elucidate the molecular mechanism underlying chondrocyte senescence mediated by Sirt6, we performed RNA-seq analysis in Sirt6 KO and WT chondrocytes. The IL-15, JAK3, and STAT5 genes were observed to be remarkably increased in Sirt6 KO chondrocytes (Fig. 4a, b and Supplementary Fig. 4a, b). The upregulated genes were significantly enriched in cell aging, mitochondrial matrix, and mitochondrion-plasma membrane adapter activity, which were referred to as the biological process (BP), cellular component (CC), and molecular function (MF), respectively. (Fig. 4c). Furthermore, GSEA and KEGG analysis demonstrated that JAK/STAT signaling pathway was markedly upregulated (Fig. 4d). It should be noted that JAK3 is activated downstream of stimulation with several cytokines, including IL-15^27,28^. Rescue experiments were further performed to validate the relationship between Sirt6 and IL-15/JAK3/STAT5 signaling pathway (Fig. 4e).

Notably, Scanzello et al.^29^ found that IL-15 was significantly elevated in synovial fluid in early knee OA and its level was closely associated with MMP1 and MMP3 expressions. Moreover, Warner et al.^30^ reported that IL-15 signaling may be a target for pain in OA patients. Subsequently, we investigated the precise molecular mechanism concerning how Sirt6 regulates IL-15/JAK3/STAT5 signaling pathway. Histone H3’s acetylated lysines, H3K9ac, H3K56ac, and H3K18ac, are the targets of Sirt6, a histone deacetylase (HDAC) that has historically been identified^31–33^. Notably, Sirt6 is a complex enzyme with multiple enzymatic processes and substrates in addition to histone deacetylation, which deacetylates several non-histone proteins implicated in genomic instability and cellular senescence^33^. Acetylation and deacetylation of STATs are a very dynamic process facilitated by the rather large number of histone acetyltransferases (HATs) and HDACs^34,35^. Sirt6 overexpression can significantly inhibit the phosphorylation of STAT5 induced by IL-15 (Fig. 4f). Further, Sirt6 overexpression attenuated the IL-15/JAK3-induced STAT5 translocation from cytoplasm to nucleus (Fig. 4g, h). To further clarify the regulatory relationship between Sirt6 and STAT5, co-immunoprecipitation was performed and the result demonstrated that Sirt6 could interact with STAT5, further enhanced by IL-15 stimulation (Fig. 4i, left). Subsequently, we determined whether STAT5 is the direct substrate for the deacetylase activity of Sirt6 using a deacetylation assay in chondrocytes. IL-15 treatment increased the acetylation of STAT5, which was abolished by the overexpression of Sirt6 (Fig. 4i, right). Luciferase reporter assay further showed that co-transfection of Sirt6 decreased the transcriptional activity of STAT5 (Fig. 4j). Further, ChIP assay demonstrated that Sirt6 reduced the recruitment of STAT5 to promoter regions of p16^INK4a^ and IL-6 (Fig. 4k) and TNF-α and MMP3 genes (Supplementary Fig. 4c). Together, these results indicate that Sirt6 could deacetylate STAT5, further affecting its phosphorylation.

### Sirt6 deacetylates STAT5 on Lysine 163 (K163)

**Fig. 5:**
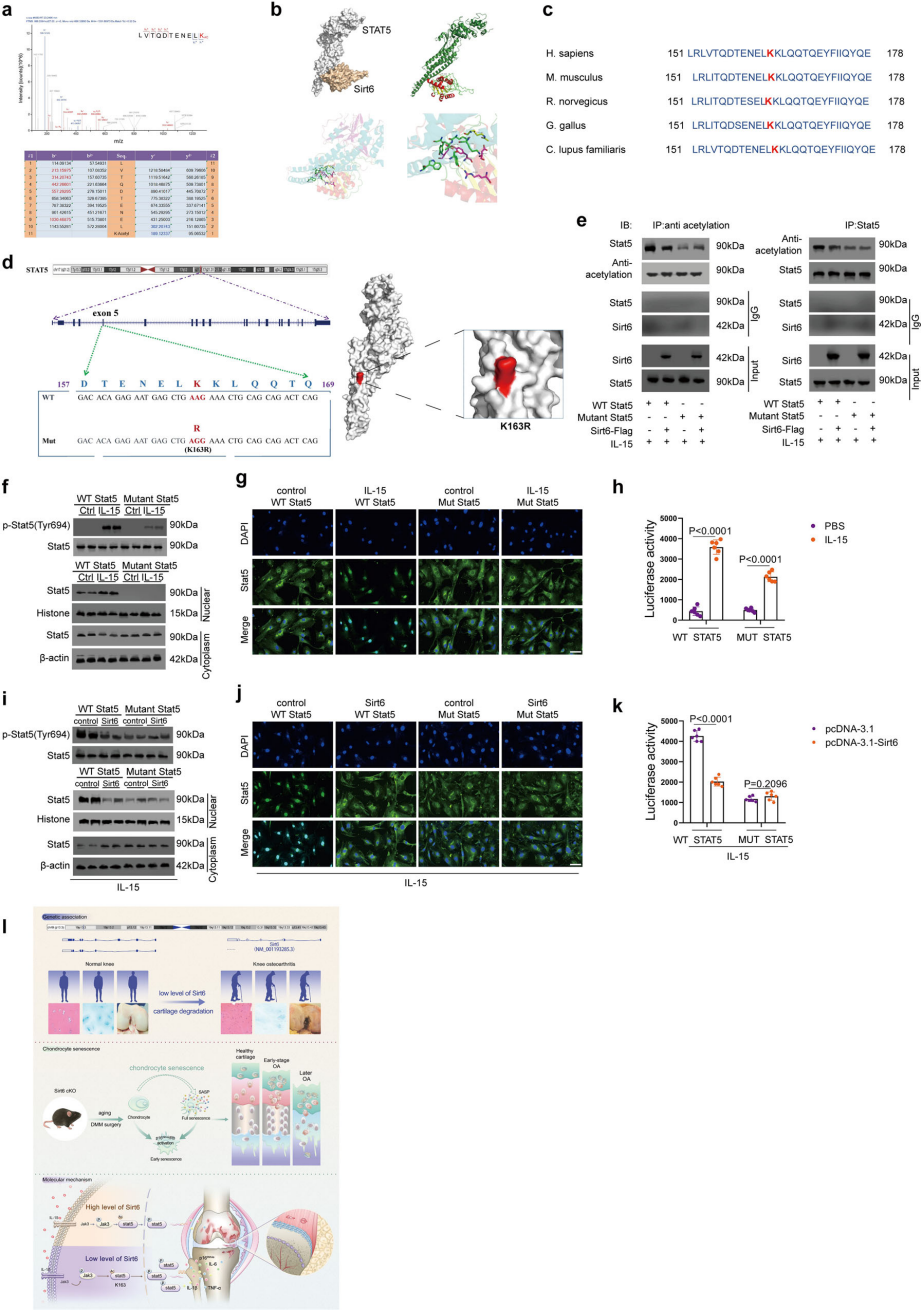
Sirt6 modulates IL-15/JAK3/STAT5 signaling pathway by deacetylating lysine 163 (K163) on Stat5. **a** LC-MS/MS analysis identified Sirt6 deacetylated K163 on Stat5. Lysates from human chondrocytes with or without Sirt6 treatment. **b** Interaction model between Stat5 and Sirt6. **c** Conservation of Stat5 K163 in different species. **d** K163 is located in exon 5 of STAT5, which was mutated to arginine through “A” replaced by “G”. The surface from molecular simulation showed the mutant site (K163R). **e** Sirt6 deacetylated Stat5 via K163. The acetylation level of WT Stat5 with Sirt6 treatment was analyzed by western blot. *n* = 3 independent biological replicates per group. **f**, **g** Human chondrocytes were transfected with WT Stat5 or mutant Stat5, followed by IL-15 treatment. The phosphorylation of Stat5 and the cellular localization of Stat5 was determined by western blot. *n* = 3 independent biological replicates per group (**f**). Immunofluorescent staining of Stat5. *n* = 3 independent biological replicates per group (**g**). **h** Transcriptional activity of WT and mutant Stat5. Human chondrocytes were co-transfected with WT Stat5 or mutant Stat5, followed by IL-15 treatment. *n* = 6 independent biological replicates per group. **i**, **j** K163 of Stat5 is required for Sirt6-regulated IL-15/JAK3/STAT5 signaling pathway. After IL-15 treatment, the phosphorylation of Stat5 and the cellular localization of Stat5 were analyzed. *n* = 3 independent biological replicates per group (**i**). Immunofluorescent staining of Stat5. *n* = 3 independent biological replicates per group (**j**). **k** The transcriptional activity of WT and mutant Stat5 in human chondrocytes transfected by pcDNA3.1 or pcDNA3.1-Sirt6, followed by IL-15 treatment. *n* = 6 independent biological replicates per group. **l** Molecular model for dysregulated Sirt6 in chondrocyte senescence and OA pathogenesis. Scar bar: **g**, **j** 20 μm. Data were presented as the mean ± s.e.m. *P* values are from two-tailed unpaired Student’s *t*-test (**h**, **k**). Source data are provided as a Source Data file.

To map the deacetylation site of STAT5 by Sirt6, we employed mass spectrometry (MS) of STAT5-overexpressing chondrocytes with or without Sirt6. K163 of STAT5 was the only amino acid that was deacetylated by Sirt6 (Fig. 5a and Supplementary Fig. 5a). The interaction between Sirt6 and STAT5 was further confirmed by molecular docking and molecular dynamics simulations (Fig. 5b and Supplementary Fig. 5b, c). Notably, K163 in STAT5 is highly conserved among different species, suggesting an important role of K163 in STAT5 (Fig. 5c). It should be noted that changing K163 lysine to arginine reduced STAT5 acetylation level and eliminated Sirt6 regulatory function (Fig. 5d, e). On a functional level, the K163 mutation decreased the IL-15-induced phosphorylation of STAT5 (Fig. 5f). Immunofluorescence labeling provides further evidence that the K163 mutation consistently reduced the nuclear-localized STAT5 (Fig. 5f). When K163 was mutated, immunofluorescence labeling revealed less STAT5 in the nucleus (Fig. 5g). A luciferase test showed that mutant STAT5 had reduced transcriptional activity (Fig. 5h). Because K163 mutation eliminated Sirt6 effect on lowering STAT5 phosphorylation and nuclear localization (Fig. 5i) and transcriptional activity, it is significant that Sirt6 needed K163 for its effects on STAT5 cellular location and transcriptional activity (Fig. 5j, k). Moreover, we found that acetylation of the lysine 163 (K163) of STAT5 exposes its Tyr 694, predisposing it to phosphorylation (Supplementary Fig. 5d). In comparison, deacetylation of the lysine 163 (K163) of STAT5 was associated with an unexposed Tyr 694 site, causing it to fail to phosphorylate (Supplementary Fig. 5d). During chondrocyte senescence, we found that acetylation and phosphorylation of STAT5 increased (Supplementary Fig. 5e). When silencing STAT5, chondrocyte senescence and osteoarthritis progression can be inhibited (Supplementary Fig. 5f, g). These findings indicate that Sirt6 regulates senescence by modulating STAT5 phosphorylation, nuclear translocation, and transcriptional activity, which ultimately inhibits IL-15/JAK3/STAT5 signaling (Fig. 5l).

### Characterization of Cy5.5 labeled-tgg2-functionalized PEGylated PAMAM-MDL-800 nanoparticle (NP)

**Fig. 6:**
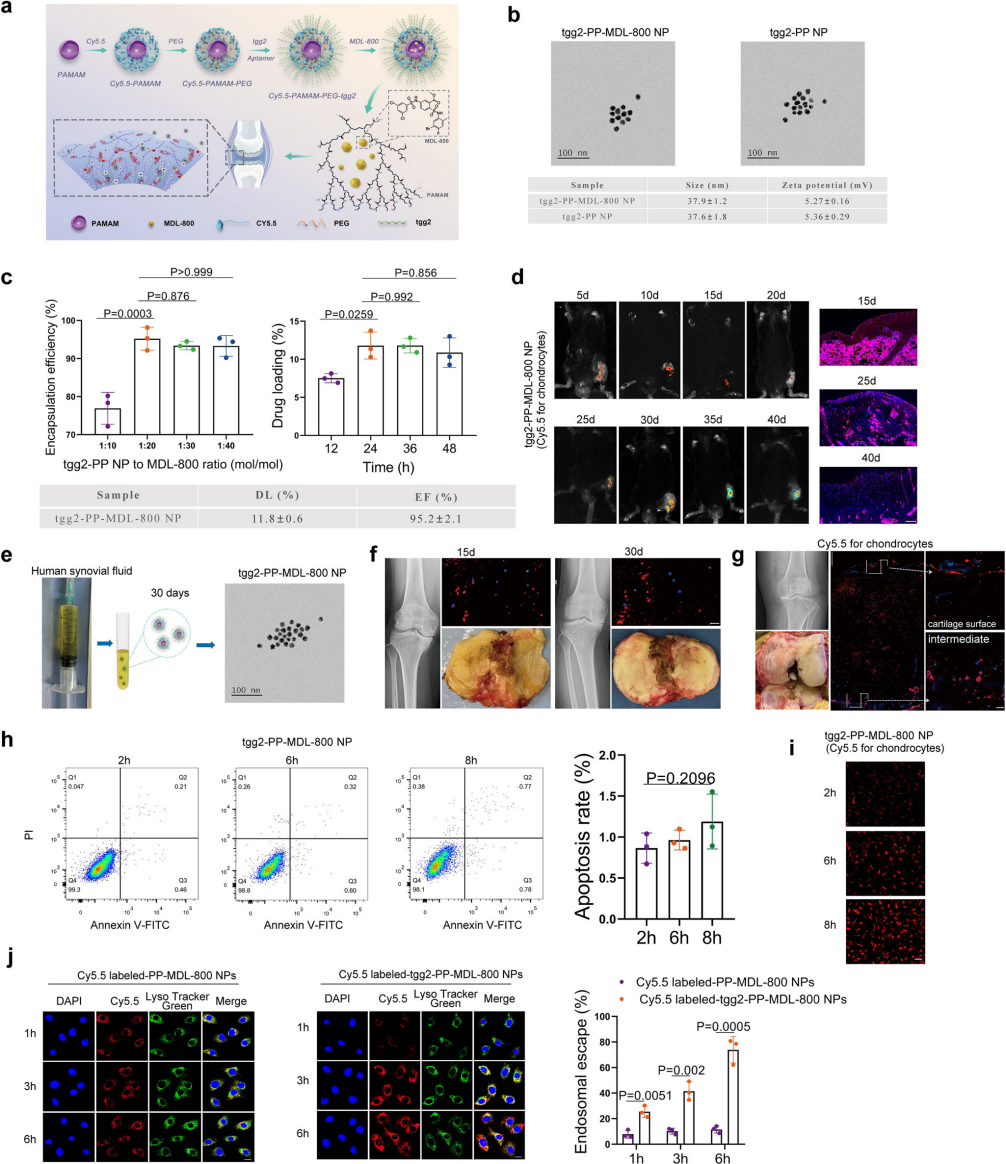
Evaluation of Cy5.5 labeled-tgg2-PP-MDL-800 nanoparticle (NP) in vitro and in vivo. **a** Schematic of Cy5.5 labeled-tgg2-PP-MDL-800 NP synthesis. **b** TEM image of the tgg2-PP-MDL-800 NP (MDL-800 to tgg2-PP NP 20:1). *n* = 3 independent biological replicates per group. **c** Effects of the MDL-800 feed ration and incubation time on drug loading (DL) and encapsulation efficiency (EF). The optimal feed ratio and incubation time were 20:1 (MDL-800 to tgg2-PP NP) and 24 h. *n* = 3 independent biological replicates per group. **d** In vivo imaging demonstrating preferential accumulation of tgg2-PP-MDL-800 NP within knee joint cavity. *n* = 6 mice per group. **e** tgg2-PP-MDL-800 NP was incubated in synovial fluid from OA patients. TEM was used to detect this NP after 30 days of incubation with synovial fluid. *n* = 3 independent biological replicates per group. **f** Human OA cartilage explants obtained from patients during surgery were incubated with fluorescent NP for 48 h, the excess NP was then washed off, and explants were kept in a complete culture medium for up to 30 days. *n* = 6 independent biological replicates per group. **g** Sagittal cartilage from OA patients' sections were examined for depth of NP penetration. *n* = 6 independent biological replicates per group. **h** The effect of tgg2-PP-MDL-800 NP on chondrocyte apoptosis was analyzed by flow cytometry at indicated time points. *n* = 3 independent biological replicates per group. **i** Cellular uptake of tgg2-PP-MDL-800 NP after 2, 6, and 8 h of incubation, respectively. *n* = 3 independent biological replicates per group. **j** Intracellular distribution of Cy5.5 labeled-PP-MDL-800 NPs and Cy5.5 labeled-tgg2-PP-MDL-800 NPs in human chondrocytes and quantification. *n* = 3 independent biological replicates per group. Scar bar: **g** (left panel) 500 μm, **d** 100 μm, **i** 50 μm, **f**, **g** (right panel), **j** 20 μm. Data were presented as the mean ± s.e.m. *P* values are from a one-way ANOVA test followed by Tukey’s post hoc (**c**, **h**) or two-tailed unpaired Student’s *t*-test (**j**). Source data are provided as a Source Data file.

To explore the potential use of Sirt6 as a therapeutic target for OA, we examined the effects of the chemical agonist of Sirt6 on chondrocyte senescence and cartilage degradation. MDL-800 was reported as a selective allosteric activator of Sirt6, which stimulates Sirt6 catalytic activity and promotes the binding affinities of the substrate to Sirt6^36^. Previously, we have successfully constructed chondrocyte-specific aptamer (tgg2)-functionalized PEGylated PAMAM nanoparticle (tgg2-PP NP), which could be used as an efficient delivery system for promoting various small molecules into chondrocytes^37^. Importantly, PAMAM has a hydrophobic core, which can physically encapsulate hydrophobic drugs^38^. Given this, we constructed Cy5.5 labeled-tgg2-functionalized PEGylated PAMAM-MDL-800 nanoparticle (tgg2-PP-MDL-800 NP) (Fig. 6a). Cy5.5 labeled-tgg2-PP-MDL-800 NP was further evaluated. The surface zeta potential and diameter of the tgg2-PP-MDL-800 NP were 5.27 ± 0.16 mV and 37.9 ± 1.2 nm, respectively (Fig. 6b). Notably, the tgg2-PP-MDL-800 NP had similar surface zeta potential and diameter to tgg2-PP NP, indicating that MDL-800 was totally encapsulated into hydrophobic cavities of PAMAM. Moreover, the drug loading (DL) and encapsulation efficiency (EF) were 11.8 ± 0.6 (%) and 95.2 ± 2.1 (%), respectively (Fig. 6c). Mice treated with tgg2-PP-MDL-800 NP demonstrated a persistent bright signal at 40 days (Fig. 6d). The tgg2-PP-MDL-800 NP were mixed with synovial fluid from OA patients and were still detected at least 30 days (Fig. 6e), indicating that tgg2-PP-MDL-800 NP may be stable in human knee joint. This was further validated by histological analysis (Fig. 6f). The human OA cartilage explants were obtained from total knee arthroplasty. Then, the cartilage explants and tgg2-PP-MDL-800 NP were co-cultured. Moreover, the tgg2-PP-MDL-800 NP can be found penetrating human OA cartilage up to a depth of at least 1500 µm (Fig. 6g), which is similar to the tgg2-PP NP reported in our previous study^37^. Chondrocyte apoptosis was analyzed using normal human cartilage tissues obtained from amputation (Fig. 6h and Supplementary Fig. 6a). High cellular uptake was observed (Fig. 6i). Moreover, histological evaluation was performed in the liver, kidney, and lungs of mice at 3 months after intra-articular (IA) injection (Supplementary Fig. 6b). Of note, the escape of small molecule from endosomal compartments to cytosol has been considered as the most important challenge. In human chondrocytes treated using Cy5.5 labeled-PP-MDL-800 NPs, very little red fluorescence dots (Cy5.5) separated from the green fluorescence (LysoTracker) were observed at 1, 3, and 6 h (Fig. 6j and Supplementary Fig. 6c). In contrast, the overlap between the green and red fluorescence was reduced in human chondrocytes treated by Cy5.5 labeled-tgg2-PP-MDL-800 NPs at all three time points (particularly 3 and 6 h) (Fig. 6j and Supplementary Fig. 6c), implicating successful escape of from endolysosomes. These results suggest that tgg2-PP-MDL-800 NPs are stable and can reach all resident chondrocytes requiring treatment for therapeutic gain.

### Pharmacological activation of Sirt6 prevents cartilage senescence and OA

**Fig. 7:**
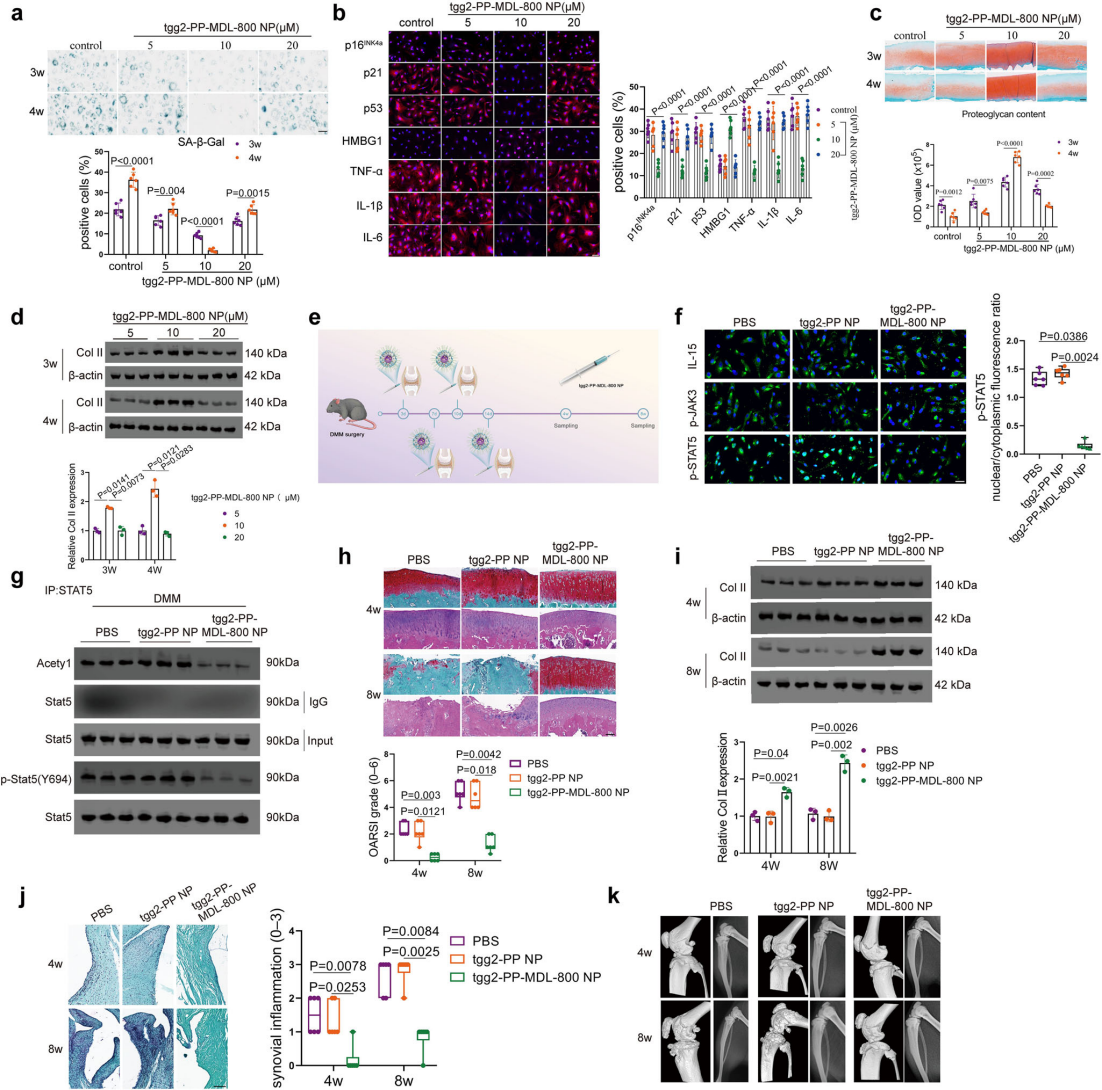
tgg2-PP-MDL-800 NP attenuates chondrocyte senescence and OA progression. **a** SA-β-Gal positivity was analyzed in human OA chondrocytes after 3 and 4 weeks of culture with tgg2-PP-MDL-800 NP or control (5, 10, or 20 µM). *n* = 6 independent biological replicates per group. **b** Representative immunofluorescent images of indicated markers in human OA chondrocytes after 4 weeks of culture with tgg2-PP-MDL-800 NP or control (5, 10, or 20 µM). *n* = 6 independent biological replicates per group. **c** Proteoglycan content was measured by safranin O-fast green staining in human OA cartilage explants after 3 and 4 weeks of incubation with tgg2-PP-MDL-800 NP. *n* = 6 independent biological replicates per group. **d** Western blot were performed to determine the Col II expression level at 3 and 4 weeks. *n* = 3 independent biological replicates per group. **e** Overview of the experimental set-up with injections of PBS, tgg2-PP NP or tgg2-PP-MDL-800 NP. **f** Representative immunofluorescent images of IL-15, p-JAK3, and p-STAT5 levels in these chondrocytes from different treatment groups. *n* = 6 independent biological replicates per group. **g** Western blot analysis of protein levels of Acetyl, p-Stat5 (Y694), and Stat5. *n* = 3 independent biological replicates per group. **h**, **i** At 4 and 8 weeks, Safranin O-fast green stained sections and H&E of cartilage (medial femoral condyle) showed that OA phenotype was significantly alleviated in OA mice model treated by tgg2-PP-MDL-800 NP, as evaluated by OARSI. *n* = 6 mice per group (**h**). A high level of Col II was observed in the tgg2-PP-MDL-800 NP treatment group. *n* = 3 independent biological replicates per group (**i**). **j** At 4 and 8 weeks, synovial inflammation was evaluated. *n* = 6 mice per group. **k** Representative micro-CT images of subchondral bone in the medial tibial plateau of PBS, tgg2-PP NP, or tgg2-PP-MDL-800 NP-treated DMM mice at 4w and 8w. *n* = 6 mice per group. Scar bar: **c** 200 μm, **h**, **j** (upper) 50 μm, **a**, **b**, **j** (lower) 20 μm, **f** 10 μm. Data were presented as the mean ± s.e.m (**a**–**d**, **i**) or median (25–75th percentiles) (**f**, h, j). *P* values are from two-tailed unpaired Student’s *t*-test (**a**, control and 10 μM groups in **c**), two-tailed unpaired *t*-test with Welch’s correction (5 and 20 μM groups in **c**), one-way ANOVA test followed by Tukey’s post hoc (**b**), two-way ANOVA test followed by Tukey’s post hoc (**d**, **i**), Kruskal–Wallis test followed by Dunn’s post hoc (**f**) or Scheirer–Ray–Hare test followed by Dunn’s post hoc (**h**, **j**). Source data are provided as a Source Data file.

Human OA chondrocytes or cartilage explants were cultured in the presence or absence of tgg2-PP-MDL-800 NP (5, 10, and 20 μM, respectively) for 3 and 4 weeks to test its efficacy. The Sirt6 level was determined at 3 and 4 weeks (Supplementary Fig. 7a). After 4 weeks of treatment, reduced senescence-associated-galactosidase (SA-β-Gal) positivity was observed in tgg2-PP-MDL-800 NP treatment group, especially in 10 μM (Fig. 7a). Further, immunofluorescence staining showed that MDL-800 significantly decreased expressions of p16^INK4a^, p21, p53, HMBG1, and SASP (TNF-α, IL-1β, and IL-6) (Fig. 7b). Of note, dramatically increased proteoglycan content was found in the tgg2-PP-MDL-800 NP group compared with that in the control group, especially at 10 μM in terms of the integrated optical density (IOD) value for the proteoglycan content (Fig. 7c), which was further confirmed by western blot (Fig. 7d). In human OA chondrocytes and IL-1β-induced mice OA chondrocytes, upregulation of the expression levels of COL2A1, ACAN and PRG4 was observed upon tgg2-PP-MDL-800 NP treatment (10 μM), indicating retention of the chondrocyte phenotype (Supplementary Fig. 7b, c).

Next, we sought to investigate whether IA injection of tgg2-PP-MDL-800 NP could attenuate OA progression after DMM in mice. The injected nanoparticles distributed inside the joint was evaluated (Supplementary Fig. 7d). A DMM model was established in WT mice, followed by IA injection of phosphate-buffered saline (PBS), tgg2-PP NP or tgg2-PP-MDL-800 NP once a week for 3 weeks. OA progression was assessed at 4 and 8 weeks post-OA surgery (Fig. 7e). Of note, activation of Sirt6 (MDL-800 treatment) inhibited IL-15/JAK3/STAT5 signaling through decreasing acetylation and phosphorylation level of STAT5 (Fig. 7f, g), which decreased senescence markers (p16^INK4a^, p21 and p53), inflammatory SASP factors (IL-1β, TNF-α, and IL-6) and cartilage-degrading enzyme level (MMP13) and increased Col II, Aggrecan, and HMBG1 levels (Supplementary Fig. 7e). Intriguingly, senescent phenotypes, decreased SA-β-Gal positivity and ROS activity, were found in tgg2-PP-MDL-800 NP group (Supplementary Fig. 7f, g). By contrast, in DMM-operated mice, IA injection of tgg2-PP-MDL-800 NP remarkably protected the structure of articular cartilage, maintained proteoglycan in the cartilage, and decreased synovitis inflammation (Fig. 7h–j and Supplementary Fig. 7h, i). We further investigated the effect of IA injection of tgg2-PP-MDL-800 NP on cartilage damage, osteophyte development, and subchondral bone remodeling in DMM mice using X-ray and micro-CT. As shown in Fig. 7k, tgg2-PP-MDL-800 NP remarkably decreased the osteophyte formation and maintained joint space compared with PBS and tgg2-PP NP at 8w. Moreover, tgg2-PP-MDL-800 NP treatment suppressed the tibial subchondral bone remodeling with decreased trabecular bone volume per total volume (BV/TV) and trabecular bone pattern factor (Tb. Pf) compared with PBS and tgg2-PP NP (Supplementary Fig. 7j). In pain-related behavioral tests, mice receiving tgg2-PP-MDL-800 NP injection exhibited higher pain thresholds (Supplementary Fig. 7k), suggesting that MDL-800 not only ameliorated histological features, but also reduced pain, a prominent symptom observed in OA patients. Collectively, these findings imply that targeting senescence for OA therapy is a promising new approach.

## Discussion

In the present study, we show that Sirt6 was markedly downregulated in cartilage tissues and chondrocytes from OA patients, and its level was strongly correlated with cartilage degradation grade. Using both loss- and gain-of-function models and Sirt6 cKO mice, Sirt6 was found to be a crucial regulator in cartilage development and chondrocyte senescence. Mechanistically, Sirt6 physically interacted with STAT5 and deacetylated its K163, which attenuates the IL-15/JAK3-induced STAT5 translocation from cytoplasm to the nucleus, ultimately inhibiting IL-15/JAK3/STAT5 signaling. More importantly, mutation of STAT5 on K163 abolished the regulatory effect of Sirt6 on STAT5 transcriptional activity. Pharmacological activation of Sirt6 by IA delivery of MDL-800 protected mice against OA.

To the best of our knowledge, the present study is the first to show the essential role of Sirt6 in embryonic chondrocyte senescence and cartilage development using Sirt6 cKO mice. Cellular senescence is a form of cell-cycle arrest implicated in embryonic growth and patterning^25,39^. Embryonic senescent cells are nonproliferative and share features with oncogene-induced senescence, including expression of p21, p53, and mediators of SASP^32^. Of note, mice deficient in p21 have defects in embryonic senescence, apical ectodermal ridge maintenance, and patterning^26^. In this study, we demonstrated that P21 and P53 levels were significantly downregulated at E13.5, E14.5, E16.5, and E18.5 of Sirt6 cKO mice compared with Sirt6^flox/flox^. Notably, senescence biomarkers decreased in Sirt6 cKO mice during embryonic chondrocyte senescence, which is contradictory to those in adult chondrocyte senescence. This may be due to other signaling pathways involved in embryonic chondrocyte senescence and cartilage development. IL-15/JAK3/STAT5 pathway may play a unique role in adult chondrocyte senescence in the context of OA condition. Importantly, the relationship between the PI3K/FOXO and TGF-β/SMAD pathways and the p21 in embryonic senescence has been extensively investigated^40,41^. Cells with a high level of PI3K/FOXO showed a low level of p21, and conversely, high levels of p21 were associated with low level of PI3K/FOXO. Additionally, a low level of TGF-β inactivates the transcription of the p21 gene through SMAD complexes, which may account for the decrease of senescence markers p21 and p53 in embryonic chondrocyte senescence. These populations of cells in the embryo express a set of markers of diverse cellular functions that, for some reason, may be reactivated upon damage or aging in adult cartilage tissues. Sirt6 may participate in chondrocyte senescence through different regulatory mechanisms in different stages (embryo and adult). The size of the Sirt6 cKO mice skeleton was smaller than those of Sirt6^flox/flox^ embryos. Furthermore, an increased percentage of the hypertrophic zone was observed in the limb of Sirt6 cKO mice. With respect to these important observations, future studies should specifically address the molecular mechanism underlying the dysregulation of P21 and P53 in Sirt6 cKO mice. Taken together, these findings highlight the critical role of Sirt6 in regulating chondrocyte senescence and cartilage development during the embryo.

Classically, upon engagement by IL-15, receptor-associated JAK3 become activated and phosphorylate both each other and the intracellular tail of their receptors, thereby creating docking sites for latent, cytoplasmic transcription factors. JAK3-mediated phosphorylation activates STAT5, which in turn directly binds DNA and regulates gene expression^42,43^. Recent studies showed that the acetylation of STAT5 also plays an important role in this pathway^44,45^. For STAT5, lysine 681, lysine 694, lysine 696, lysine 699, and lysine 705, etc., have been observed for efficient acetylation. In this study, mass spectrometry revealed a novel acetylation of STAT5, i.e., K163, in OA chondrocytes, which can be deacetylated by Sirt6. Sirt6 overexpression significantly inhibited the IL-15/JAK3-induced STAT5 translocation from the cytoplasm to the nucleus, which inactivates the IL-15/JAK3/STAT5 signaling. The relationship between acetylation and phosphorylation has been reported in several previous studies^46–48^. To date, there is no report on STAT5 expression and mutation in K163 or phosphorylation site and their association with OA onset and progression. The present study is the first to demonstrate the critical role of mutation in K163 of STAT5 in the OA phenotype. Studies focusing on K163 of STAT5 may provide a new perspective for OA treatment.

Recently, Meng et al.^49^ reported that Sirt1 can deacetylate Sirt6 at K33, which is important for Sirt6 polymerization and mobilization toward DSBs. K33-deacetylated Sirt6 anchors to γH2AX, allowing its retention on and subsequent remodeling of local chromatin. Interestingly, Sirt1 was found to be significantly dysregulated in this study. Based on these important findings, K33 or other lysine sites in Sirt6 may be deacetylated by Sirt1 in chondrocytes in the context of chondrocytes senescence and osteoarthritis. The non-histone protein deacetylation by Sirt1 is involved in key cellular processes relevant to physiology and disease, such as gene transcription, DNA damage repair and metabolism, etc.^50,51^. Therefore, the effect of K33 or other lysine sites deacetylation by Sirt1 on Sirt6 protein function should be further investigated in senescent chondrocytes in the future study.

A growing body of evidence suggests that chondrocyte senescence is potentially a common molecular mechanism that drives or promotes both age-associated and post-traumatic OA^52–55^. We demonstrated that Sirt6 deficiency in mice promoted age-related and trauma-induced chondrocyte senescence and OA development. In comparison, IA injection of Ad-Sirt6 substantially attenuated DMM-induced OA. Further investigation of the targeted activation of Sirt6 for OA treatment is thus of great value. MDL-800 is a first-in-class small-molecule cellular Sirt6 activator that can be used to physiologically and pathologically investigate the roles of Sirt6 deacetylation, which was originally developed to treat various types of cancer^36,56,57^. Our results showed that OA-related phenotypes could be significantly alleviated by targeting Sirt6 with MDL-800. Moreover, the STAT5 nuclear translocation induced by IL-15/JAK3 was also inhibited by MDL-800. This is the first study of the role of Sirt6 in cartilage senescence and OA, and the use of a selective allosteric activator of Sirt6 (MDL-800), which was encapsulated into the hydrophobic cavity of PAMAM, may be successfully Supplementary to its clinical practice in the future. Mid- and late-stage OA causes synovial oxygen tensions to drop to very low levels, which then causes the cartilage matrix’s oxygen tension to decrease as well. Due to severely impaired acid extrusion, the matrix pH is noticeably more acidic in this situation^58–60^. The polyamidoamine (PAMAM) primary and secondary amines display improved protonated action in this acidic environment, which encourages the proton sponge effect and dramatically aids the successful endo/lysosomal escape of the encapsulated MDL-800. Before being used in the clinic, MDL-800 transport kinetics must be further studied in bigger animal models with thicker cartilage that are more comparable to humans (such as pigs, cows, monkeys, etc.).

Our research demonstrated that overexpressing Sirt6 can dramatically inhibit JAK3/STAT5-dependent transcriptional activity, IL-15-induced OA, and senescence of chondrocytes. However, when K163 in STAT5 was changed to arginine, these effects were lessened. Thus, we clarified the molecular mechanism of Sirt6 in cartilage degradation, at least in part, was due to the inhibitory role of its effect on IL-15/JAK3/STAT5-dependent transcriptional activity. The deacetylase activity of Sirt6 is essential for its protective role in OA.

In conclusion, the findings presented here expands our knowledge on the mechanisms by which disruption in IL-15/JAK3/STAT5 signaling contributes to chondrocyte senescence and OA progression. Strategies that preferentially activate Sirt6 in chondrocytes may represent novel and effective approaches to prevent and treat OA, which will hopefully reduce the immense burden of this very prevalent disease.

## Methods

### Human subjects

Articular cartilage samples were sourced from 90 knee OA patients (83 females and 7 males; a median age of 61 years) who underwent total knee arthroplasty. As controls, 60 specimens (56 females and four males; a median age of 60 years) were obtained from individuals undergoing amputation surgery due to atherosclerotic necrosis of the lower extremity or diabetic lower extremity necrosis or traumatic necrosis of the lower extremity. The two groups (OA and NC) were also matched for BMI (median, 24.5 vs. 23.95). These OA patients had an average course of 6.6 years. Preoperatively, all the patients underwent a knee X-ray examination. Upon separation of cartilage from bone tissue, the cartilage was immediately snap-frozen in liquid nitrogen. The cartilage tissues were further processed for histological examination and were categorized according to the modified Mankin scoring system, i.e., grade I mild OA (Mankin scores 0–5), grade II moderate OA (Mankin scores 6–10), and grade III severe OA (Mankin scores 11–14)^61^. There were 25 grade II moderate OA and 65 grade III severe OA in this study. This study protocol was approved by the ethics committee of Zhongda hospital, and full written consent were obtained before the operative procedure.

### Mice

Sirt6^flox/flox^ and Col2a1-CreER^T2^ were purchased from Jackson Laboratories (Bar Harbor, ME, USA). To generate Col2a1-CreER^T2^;Sirt6^flox/flox^ mice, Sirt6^flox/flox^ mice were mated with Col2a1-CreER^T2^ mice to produce Col2a1-CreER^T2^; Sirt6^flox/+^ mice, which were then mated with Sirt6^flox/flox^ mice. All mice were housed under pathogen-free conditions with five or fewer mice per cage. Mice had free access to food and water. Animals were maintained under constant temperature (23–25 °C), circulating air, and humidity (45–65%) with a 12 h:12 h light/dark cycle. The male mice used for all experiments were randomly assigned to control or treatment groups and to those used in OA evaluation. The number of animals in each group is specified in figure legends. The experimental protocol was approved by and performed in accordance with protocols from the Institutional Animal Care and Use Committee of Southeast University. We have complied with all relevant ethical regulations for animal tests and research. Animal experiments were performed in accordance with appropriate international guidelines (ARRIVE; http://www.nc3rs.org.uk/arrive-guidelines).

### Senescence-associated β-galactosidase (SA-β-Gal) assay

SA-β-gal staining was performed using an SA-β-Gal staining kit (#9860, Cell Signaling Technology) as previously described methods^62,63^. Briefly, human chondrocytes or chondrocytes from Sirt6^flox/flox^ and Col2a1-CreER^T2^ were seeded onto six-well culture plates at a density of 1.0 × 10^5^ cells per well and cultured for 48 h at 37 °C in an incubator under 20% O2/5% CO_2_. After the cells had been cultured for 48 h, pcDNA3.1-Sirt6, Sirt6 siRNA, or corresponding controls were transfected by Lipofectamine® 3000 Transfection Reagent (Invitrogen, Life Technologies, Carlsbad, CA, USA). Forty-eight hours after lipofection, cytochemical staining for SA-β-Gal was performed at pH6, and the positive cells were counted.

### γH2AX foci and telomere dysfunction-induced foci (TIFs)

To detect DNA damage and telomere dysfunction, the γH2AX foci and TIFs were analyzed 48 h after lipofection. In brief, human chondrocytes were seeded onto coverslips and cultured for 24 h at 37 °C in an incubator under 20% O2/5% CO_2_. After chondrocytes had been cultured for 24 h, pcDNA3.1-Sirt6, siRNA Sirt6, or corresponding controls were transfected by lipofection. Forty-eight hours after lipofection, chondrocytes were fixed for 10 min with 4% paraformaldehyde in PBS, followed by permeabilization with 0.25% Triton X-100 in PBS for 10 min. The cells were subsequently washed with PBS, blocked for 1 h with 1% BSA (Sigma-Aldrich) in PBS containing 0.1% Tween-20, and then incubated with an anti- TRF-1 mouse monoclonal antibody (TRF-78, ab10579, Abcam, 1:1000 dilution). γH2AX was detected by incubation with a rabbit polyclonal antibody (Phospho-Histone H2A.X Ser139, Cell Signaling, 1:100 dilution, #9718).

### Telomere length measurement and telomere FISH

Genomic DNA was extracted directly from human chondrocytes using a Mini Genomic DNA Kit (QIAamp DNA Mini Kits) according to the manufacturer’s protocols (Qiagen). Telomere length was determined by using an RT-qPCR method. Telomere FISH was performed by using a PNA probe (Panagene). Briefly, chondrocytes were added to six-well culture plates with glass slides and incubated at 37 °C for 2 h. Adhered cells were swollen in KCl buffer, fixed in methanol/acetic acid (3:1), rehydrated in PBS, fixed in 4% formaldehyde, and then dehydrated in a series of concentrations of ethanol. Slides were incubated with a hybridization mixture (70% formamide, 10 mM NaHPO_4_, pH 7.4, 10 mM NaCl, 20 mM Tris buffer, pH 7.5), placed on an 80 °C heating block for 5 min to denature chromosomal DNA, and incubated with the PNA probe for 2 h at room temperature. After washing, slides were mounted with Vectashield mounting medium containing DAPI (Vector Labs) and analyzed with a confocal microscope.

### ROS assay kit

Reactive oxygen species (ROS) generation in chondrocytes was assessed with 2′,7′-dichlorofluorescein diacetate (DCFH-DA) (Beyotime, China). Cells were seeded into six-well plates with 5 × 10^5^ cells/well, and pcDNA3.1-Sirt6, siRNA Sirt6, or corresponding controls was transfected for 48 h. DCFH-DA was diluted with a serum-free medium at a ratio of 1:1000, and the final concentration was 10 mmol/L. The cell culture medium was removed, and DCFH-DA was added. The cells were incubated at 37 °C for 20 min. The cells were washed three times with a serum-free medium. ROS levels were analyzed by immunofluorescence assays.

### Site-directed mutagenesis

The pUC119 vector containing the wild-type STAT5 complementary DNA (cDNA and pUC-STAT5) was established. Based on the manufacturer′s instructions, QuikChange II site-directed mutagenesis kit was used to obtain a mutant plasmid (PUC-STAT5-K163R) (Aglient). The following primers were used: 5′-GAGTCTGCTGCAGTTTCCTCAGCTCATTCTCTGTG-3′ and 5′-CACAGAGAATGAGCTGAGGAAACTGCAGCAGACTC-3′. The primers was designed using QuikChange Primer Design (https://www.agilent.com.cn/store/primerDesignProgram.jsp). After PCR, 20 μL of the reaction was digested with DpnI at 37 °C for 1 h and 10 μL was transformed into DH5 alpha Escherichia coli to prepare the mutant construct plasmids. All constructs were confirmed by sequencing.

### Cell culture and transfection

The microdissection of mouse knee OA articular cartilage (medial femoral condyle) was performed under a surgical microscope to carefully dissect only articular cartilage and avoid subchondral bone. Macroscopically affected cartilage pieces were obtained from damaged medial femoral condyle of human OA. Primary chondrocytes were prepared from the dissected articular cartilage by enzymatic digestion. Briefly, dissected articular cartilage was rinsed in PBS, and incubated at 37 °C for 15 min in trypsin-ethylenediaminetetraacetic acid, followed by digestion with 2 mg/mL collagenase at 37 °C for 2 h in Dulbecco′s modified Eagle′s medium (DMEM) containing 10% fetal bovine serum (FBS), 100 U/mL penicillin, and 100 mg/mL streptomycin under an atmosphere of 5% CO_2_. During the culture period, cells were incubated at 37 °C in a humidified atmosphere of 5% CO_2_ and 95% air, and the medium was changed every 2–3 days. First-passage chondrocytes at 85% confluence were used for all experiments. The SW1353 cell line (ATCC, HTB-94) was also used. Toluidine blue staining and immunohistochemistry for Col II were performed to characterize chondrocytes. pCDNA3.1-STAT5-wt and pCDNA3.1-STAT5-K163A were constructed for further study (Invitrogen). The Sirt6 sequence was synthesized and sub-cloned into the pCDNA3.1 (Invitrogen) vector. Overexpression of Sirt6 was achieved via pCDNA3.1-Sirt6/pCDNA3.1-flag-Sirt6 transfection, with an empty pCDNA3.1 vector used as a control (Invitrogen). Negative control siRNA or siRNA against Sirt6 (Sigma-Aldrich, St. Louis, MO, USA) was transfected into chondrocytes at a concentration of 50 nM using Lipofectamine 3000 Transfection Reagent (Invitrogen, Life Technologies, Carlsbad, CA, USA).

### RNA isolation, cDNA synthesis, and qRT-PCR

Total RNA was extracted using TRIzol reagent (Invitrogen). RNA was specifically purified with an RNAeasy Mini Kit (Qiagen). Then, RNA quantity and quality were further determined using a nanodrop (Thermo Scientific, Waltham, MA, USA) and Bioanalyzer (Agilent Inc., Santa Clara, CA, USA). RNA was then reverse-transcribed using the PrimeScript RT Reagent Kit (Takara Bio). Quantitative polymerase chain reaction (PCR) amplification was performed with an ABI QuantStudio 5 (Applied Biosystem, Foster City, CA). The samples were run in triplicate and normalized to GAPDH using a ΔΔCt cycle threshold-based algorithm. The primer sequences used in this study are as follows: Sirt6 (human), forward: GCAGTCTTCCAGTGTGGTGT, reverse: GATAGAGCCGTTGATCCGGG; Sirt6 (mice), forward: AGGAACCTGGGTCAGGGAAG, reverse: GGAGGACTGCCACATTAGCC; GAPDH (human), forward: GGAGCGAGATCCCTCCAAAAT, reverse: GGCTGTTGTCATACTTCTCATGG; GAPDH (mice), forward: AGGTCGGTGTGAACGGATTTG, reverse: GGGGTCGTTGATGGCAACA

### Flow cytometry

Apoptosis was evaluated by staining cells with both Annexin V-FITC and PI according to the manufacturer′s instructions. Cells that were positively stained with Annexin V-FITC and negatively stained for PI were considered apoptosis. Cells that were positively stained for both Annexin V-FITC and PI were considered necrosis. The cells were stained with 5 µL Annexin V-FITC and 10 µL PI and then analysed. Analyses were performed using FlowJo Flow Cytometry Analysis Software (Tree Star).

### Chromatin immunoprecipitation (ChIP)

Chondrocytes were infected with adenovirus (Ad)-Sirt6 for 24 h, then treated with IL-15 (2 ng/mL) for 6 h. The next steps were performed with the EZ-Magna ChIP G Kit (Millipore, Billerica, MA). Purified DNA was subjected to RT-qPCR.

### Co-immunoprecipitation (co-IP)

Using a cytoplasmic and nuclear protein isolation kit (Beyotime, P0028), nuclear extracts were obtained from chondrocytes. Chondrocytes were then lysed with non-denaturing NP-40 lysis buffer supplemented with 1 mM phenylmethylsulfonyl fluoride (PMSF), protease inhibitor cocktail, and phosphatase inhibitor cocktail (Sigma). An amount of 500 μg protein was immunoprecipitated with anti-Sirt6 (Cell Signaling Technology) or anti-total-STAT5 antibody (Cell Signaling Technology) and Protein A/G PLUS-Agarose (sc-2003, Santa Cruz Biotechnology) overnight at 4 °C. Beads with the immune-precipitated complex were washed with lysis buffer three times before denaturing in lysis buffer at 97 °C for 10 min. Nuclear extracts and immunoprecipitated protein were resolved by SDS-PAGE. Western blot was then performed.

### Luciferase assay

Chondrocytes were seeded in 48-well plates and transiently co-transfected with a luciferase reporter and STAT5 with or without Sirt6. After 48 h of transfection, chondrocytes were stimulated with IL-15 (2 ng/mL) for 4 h, and cells were harvested to detect luciferase.

### RNA-sequencing analysis

Total RNA was extracted with TRIzol (Invitrogen) from Sirt6-deficient or control cells (12-week-old Sirt6 cKO and WT mice). The purity and quantity of total RNA were measured by Nanodrop. The integrity of RNA was evaluated using the RNA Nano6000 Assay Kit on the Bioanalyzer 2100 system (Agilent Technologies, CA, USA). A total of 1 μg RNA per sample was used as an input for further analysis. Strand-specific RNA-sequencing libraries were generated using the NEBNext Ultra II RNA Library Prep Kit (Illumina, USA). Library quality was evaluated on the Agilent Bioanalyzer 2100 system (Agilent Technologies, CA, USA). Final libraries were sequenced on Illumina NovaSeq 6000 platform by 150 bp paired-end reads. GO enrichment analysis was implemented by the GOseq R Package and DAVID online tool (https://david.ncifcrf.gov/). Pathways of differentially expressed genes were analyzed by the KEGG database (http://www.kegg.jp/kegg/) and GESA software.

### Mass spectrometry analysis

Chondrocytes were transfected with STAT5 with or without Sirt6, then 48 h later were treated with IL-15, and the nuclear fraction was prepared by using a kit (Beyotime, P0028). The nuclear protein was immunoprecipitated with an anti-acetylation antibody and Protein A/G PLUS-agarose (sc-2003, Santa Cruz Biotechnology) overnight at 4 °C. Immunoprecipitated protein was resolved by 10% SDS-PAGE gels for Coomassie blue staining. The purified protein bands were cut out and digested with trypsin. LC-MS/MS analysis was performed on an EASY-nLC 1000 HPLC system (Thermo Scientific), which was directly interfaced with a Q Exactive mass spectrometer (Thermo Scientific). The analytical column was an AcclaimR PepMap RSLC column (50μm ID, 15 cm length, C18, 2 μm, 100 Å) (Thermo Scientific). The Q Exactive mass spectrometer was operated in the data-dependent acquisition mode using Xcalibur 2.2 SP1 software and there was a single full-scan mass spectrum in the orbitrap (300–2000 m/z, 70,000 resolution) followed by 20 data-dependent MS/MS scans at 27% normalized collision energy (HCD). The MS/MS spectra from each LC-MS/MS run were searched against the fasta files using Sequest HT and phosphoRS 3.0 modules in Proteome Discoverer software (Version PD1.4, Thermo Scientific, USA).

### Molecular docking and molecular dynamics simulations

Sirt6-STAT5 docking was performed by ZDOCK using the default setting. The pose with the highest score was further refined by MD using the amber18 package. The ff14SB force field was used for the protein, and sodium ions were added to neutralize the negative charges in the tleap utility. The simulation system was immersed in a truncated octahedral box of TIP3P water, Supplementary 9 Å outside the protein on all sides. To begin the simulation, the complex was treated as follows: (a) water molecules and counter ions were relaxed to minimize energy during 10,000 minimization steps (8000 steepest descent steps, SD, and 2000 conjugate-gradient steps, CG) with the receptor and ligand restrained with force constant of 1500 kcal mol^-1^ Å^−2^; (b) the whole system was then minimized without restraints during 10,000 minimization steps (5000 SD and 5000 CG). After energy minimization, the system was gradually heated in the NVT ensemble from 0 to 300 K over 50 ps using the Berendsen coupling algorithm. This procedure was followed by 50 ps of NPT simulation at 300 K and 1 atm pressure using the Langevin dynamics algorithm. After equilibration, a 1000 ps production MD simulation was performed. All visualization analysis was performed in pymol1.7.

### Immunoblotting

Chondrocytes or cartilage tissues were harvested and lysed in RIPA buffer (50 mM Tris-HCl pH 7.4, 150 mM NaCl, 0.1% SDS, 1% Triton X-100, 1% Nonidet P-40, 1% sodium deoxycholate, 1 mM EDTA), added with protease inhibitor cocktail (Roche) and phosphatase inhibitor (Roche). The BCA Protein Assay Kit (Thermo Fisher Scientific) was then used to determine the protein concentrations. Total protein was subjected to SDS-PAGE gels (6–15%) and then transferred to the PVDF membrane (Millipore). The membranes were blocked in 5% skim milk for 2 h and then membranes were incubated overnight at 4 °C with primary antibodies against Sirt1 (1:1000, Cell Signaling Technology, #8469), Sirt2 (1:1000, Cell Signaling Technology, #12672), Sirt3 (1:1000, Cell Signaling Technology, #2627), Sirt4 (1:500, Santa Cruz Biotechnology, sc135797), Sirt5 (1:1000, Cell Signaling Technology, #8779), Sirt6 (1:1000, Cell Signaling Technology, #12486), Sirt7 (1:1000, Cell Signaling Technology, #5360), p16^INK4a^ (1:1000; Abcam, ab270058), IL-6 (1:500, Abcam, ab6672), IL-15 (1:1000, Abcam, ab134177), JAK3 (1:2000, Abcam, ab45141), p-JAK3 (1:1000, Cell Signaling Technology, #5031), STAT5 (1:1000, Cell Signaling Technology, #25656), p-STAT5 (1:1000, Cell Signaling Technology, #4322), Col2A1 (1:500, Abcam, ab34712), ACAN (1:1000, Abcam, ab36861), PRG4 (1:1000; Abcam, ab28484), Histone (1:1500, Abcam, ab1791), GAPDH (1:2000, Abcam, ab8245), and beta-actin (1:1000, Cell Signaling Technology, #4967). The membranes were incubated with horseradish peroxidase (HRP)-linked anti-rabbit IgG (1:1000, Cell Signaling Technology, #7074) or HRP-linked anti-mouse IgG (1:1000, Cell Signaling Technology, #7076) for 2 h. Immunocomplexes were visualized through chemiluminescence using an ECL kit (Amersham Biosciences). The bands were detected with iBright FL1000 (Thermo Scientific). Protein band intensity was quantified by densitometric analysis using ImageJ software and normalized to the corresponding bands. The uncropped blots are provided in Supplementary Figs. 8–10.

### Cell immunofluorescence

For immunofluorescence staining, chondrocytes were cultured on glass coverslips in 24-well plates and then transfected with indicated plasmids. After 24 h transfection, the cells on coverslips were washed twice with PBS and immobilized by 4% PFA for 30 min at room temperature. After washing three times with PBS (5 min each time), cells were blocked by blocking solution (5% BSA in TBS with 0.5% Triton X-100) for 2 h for the following incubation with primary antibodies, p16^INK4a^ (1:1000, Cell Signaling Technology, #18769), TNF-α (1:500; Abcam, ab1793), IL-6 (1:1000; Abcam, ab246703), IL-1β (1:100, Abcam, ab156791), P21 (1:800, Cell Signaling Technology, #2947), p53 (1:1000, Cell Signaling Technology, #2527), HMBG1 (1:2000; Abcam, ab18256), p-JAK3 (1:1000; Abcam, ab45141), STAT5 (1:200, Cell Signaling Technology, #25656), p-STAT5 (1:100, Cell Signaling Technology, #4322) and Alexa Fluor 555 (1:100, Abcam, ab150078)- or Alexa Fluor 488 (1:1000, Abcam, ab150077)-conjugated secondary antibodies and DAPI (Invitrogen). The fluorescence was visualized under CarlZeiss LSM710 confocal microscope (CarlZeiss, Oberkochen, Germany). For quantifying endosomal escape, the percentage of positive cells (red and green) was calculated by Image-Pro Plus 6.0 software. Finally, the ratio of red immunofluorescence signals to green immunofluorescence signals was calculated, i.e., endosomal escape (%).

### Immunohistochemistry (IHC), immunofluorescence staining, and TUNEL

According to the manufacturer’s protocol of the Histostain-Plus IHC Kit (Invitrogen, USA), slides were quenched in 3% H_2_O_2_ in methanol and rinsed three times in PBS after deparaffinization and rehydration. The slides were then subjected to antigen retrieval using Trypsin for 20 min at 37 °C. After washing three times in PBS, the slides were incubated with a blocking reagent for 30 min. The slides were incubated with primary antibodies, p16^INK4a^ (1:1000; Abcam, ab241543), TNF-α (1:500; Abcam, ab220210), IL-6 (1:100; Abcam, ab290735), Col II (1:200; Abcam, ab34712), ACAN (1:500; Abcam, ab186414), Col X (1:1000; Abcam, ab49945), p21 (1:50, Cell Signaling Technology, #2947), p53 (1:100, Cell Signaling Technology, #48818), IL-1β (1:200, Cell Signaling Technology, #12242) and HMBG1 (1:400; Abcam, ab79823), biotinylated secondary antibodies, enzyme-conjugated substrate and developed with diaminobenzidine (DAB) chromogen. For immunofluorescence staining, paraffin sections were microwave pretreated, incubated with primary antibodies, incubated with secondary antibodies, and counterstained with DAPI in mounting media. Terminal deoxynucleotidyl transferase (TdT) dUTP Nick-End Labeling (TUNEL) assay was performed according to the manufacturer′s instructions (In Situ Cell Death Detection Kit, Fluorescein, Roche).

### Preparation of tgg2-functionalized PEGylated PAMAM-MDL-800 nanoparticle

Based on our previously constructed NP system (tgg2-functionalised PEGylated PAMAM nanoparticle, tgg2-PP NP)^35^, MDL-800 was encapsulated into the tgg2-PP NP. A total of 3 mg of MDL-800 was dissolved in 5 ml methanol. A certain amount of tgg2-PP NP was weighed according to the molar ratio of MDL-800 to tgg2-PP NP 20:1. The tgg2-PP NP was then added to the methanol solution. Protected by nitrogen, the mix was stirred for 24 h in a 37 °C dark environment. After the removal of the methanol, an appropriate amount of distilled water was added to dissolve the tgg2-PP NP/MDL-800 complex. Subsequently, the complex solution was transferred to an ultrafiltration tube and centrifuged at 10000 rpm for 30 min for removal of uncoating MDL-800. The powder obtained by freezing and drying of supernatant is tgg2-PP-MDL-800 NP. Dynamic light scattering (DLS) and transmission electron microscopy were used to evaluate the complex.

### Drug loading and encapsulation efficiency evaluation

Ultrafiltration centrifugation method was used to determine the drug loading (DL) and encapsulation efficiency (EF) of the compound (tgg2-PP-MDL-800 NP). The compound solution was placed in an ultrafiltration centrifuge tube and centrifuged at 10000 rpm for 30 min. After dilution of the filtrate, the fluorescence intensity was determined by a fluorescence spectrophotometer at 558 nm, and the amount of free drugs was calculated. According to the following formula, drug loading and encapsulation efficiency were calculated.

$${{{{{\rm{DL}}}}}}(\%)=\frac{{{{{{\rm{The}}}}}}\,{{{{{\rm{total}}}}}}\,{{{{{\rm{amount}}}}}}\,{{{{{\rm{of}}}}}}\,{{{{{\rm{drug}}}}}}\,{{{{{\rm{put}}}}}}\,{{{{{\rm{in}}}}}}-{{{{{\rm{free}}}}}}\,{{{{{\rm{drug}}}}}}\,{{{{{\rm{dose}}}}}}}{{{{{{\rm{The}}}}}}\,{{{{{\rm{total}}}}}}\,{{{{{\rm{amount}}}}}}\,{{{{{\rm{of}}}}}}\,{{{{{\rm{drug}}}}}}\,{{{{{\rm{put}}}}}}\,{{{{{\rm{in}}}}}}+{{{{{\rm{the}}}}}}\,{{{{{\rm{amount}}}}}}\,{{{{{\rm{of}}}}}}\,{{{{{\rm{NP}}}}}}}{{{{{\rm{X}}}}}}\,100\%$$

$${{{{{\rm{EF}}}}}}(\%)=\frac{{{{{{\rm{The}}}}}}\,{{{{{\rm{total}}}}}}\,{{{{{\rm{amount}}}}}}\,{{{{{\rm{of}}}}}}\,{{{{{\rm{drug}}}}}}\,{{{{{\rm{put}}}}}}\,{{{{{\rm{in}}}}}}-{{{{{\rm{free}}}}}}\,{{{{{\rm{drug}}}}}}\,{{{{{\rm{dose}}}}}}}{{{{{{\rm{The}}}}}}\,{{{{{\rm{total}}}}}}\,{{{{{\rm{amount}}}}}}\,{{{{{\rm{of}}}}}}\,{{{{{\rm{drug}}}}}}\,{{{{{\rm{put}}}}}}\,{{{{{\rm{in}}}}}}}{{{{{\rm{X}}}}}}\,100\%$$

### Cellular uptake and intracellular distribution

Chondrocytes were treated with tgg2-PP NP or tgg2-PP-MDL-800 NP at 37 °C for up to 6 h, then the chondrocytes were incubated at 37 °C for 1 h with 50 nM DAPI and 100 nM LysoTracker Green in cell culture medium. The cells were then fixed with 4% paraformaldehyde and examined (FluoView FV1000 confocal microscope, Olympus).

### The establishment of the mouse OA model and MDL-800 treatment

Eight-week-old Col2a1-CreER^T2^;Sirt6^flox/flox^ and Sirt6^flox/flox^ mice (male, C57BL/6) were injected intraperitoneally with tamoxifen (TM) (100 μg/g body weight, Sigma, St. Louis, MO, USA) daily for 5 days. For the experimental OA model, a surgical procedure was performed in 10-week-old mice. Under general anesthesia (with ketamine and xylazine), DMM was surgically performed by transection of the medial meniscotibial ligament in the right knee joints using a surgical microscope. Sham operations were also performed by opening and exposing the structures of the right knee and then closing the skin incision without disturbing the joint tissue. For the aging experiments, we compared Col2a1-CreER^T2^;Sirt6^flox/flox^ and Sirt6^flox/flox^ mice at 6, 12, and 18 months. For MDL-800 treatment of experimental OA, 15 μL volume PBS, tgg2-PP NP, or tgg2-PP-MDL-800 NP (10 μM) were injected into the knee joint using a 33 G needle and a micro-syringe (Hamilton). The mice received the first injection 3 days after DMM. Mice were sacrificed 4 and 8 weeks after treatment and subjected to histopathological and radiographic analysis. Testing for mechanical allodynia (von Frey sensitivity) was performed using a calibrated set of von Frey filaments (Stoelting, Wood Dale, IL).

### Micro-CT, X-ray, and histological evaluation

According to the methods described in our previous study^36^, the mouse joints were scanned using the Skyscan 1176 micro-CT scanner (Skyscan, Aartselaar, Belgium). Image slices were reconstructed using NRecon software (version 1.6.3.2, Skyscan). The following morphometric parameters, trabecular bone volume fraction (BV/TV) and trabecular bone pattern factor (Tb. Pf) were determined using CT Analyzer software (SkyScan). Radiographs of mouse knee joints were obtained using the Faxitron MX20 X-ray system. For whole skeletal staining, skeletons of whole-mouse embryos were stained with Alcian blue and Alizarin red (Sigma-Aldrich). hematoxylin and eosin (H&E), Alcian blue, safranin O/fast green, Masson′s trichrome, and immunohistochemical analysis were performed. The cartilage degradation grade (OARSI grading system, grade 0–6)^64,65^, synovitis (grade 0–3)^66^, and osteophyte maturity^67^ were quantified as described previously. The histological images were acquired using a DS-Ri2 camera (Nikon).

### Statistical analysis

All statistical analyses were performed using GraphPad Prism 8 (GraphPad Software Inc., La Jolla, CA, USA) and R software (version 4.2.1). Data were presented as the mean ± s.e.m or median (25–75th percentiles), as indicated in figure legends. The Kolmogorov–Smirnov and Shapiro–Wilk tests were performed to determine the data distribution. For normally distributed data, an unpaired *t*-test or unpaired *t*-test with Welch’s correction was used to compare two independent groups. In multiple comparisons, one or two-way-ANOVA followed by Tukey’s post hoc test or Brown–Forsythe ANOVA test followed by Tamhane’s T2 post hoc analysis was used. For non-normally distributed data, Mann–Whitney *U-*test was used to compare two independent samples. Kruskal–Wallis test was performed for multiple comparisons, followed by Dunn’s post hoc test. A non-parametric equivalent of the two-way ANOVA Scheirer–Ray–Hare test was performed for related samples’ comparison, followed by Dunn’s post-test. Spearman’s correlation analysis was performed between the Sirt6 level and the modified Mankin scale and synovitis. A *p* value less than 0.05 was considered statistically significant. All statistical tests used were two-sided.

### Reporting summary

Further information on research design is available in the Nature Portfolio Reporting Summary linked to this article.

## Supplementary information

Supplementary Information

Reporting Summary

## Data Availability

The original RNA-seq data generated in this study (three control and three Sirt6 KO mice chondrocytes) have been deposited in the GEO database under accession code GSE206513. All other relevant data supporting the findings of this study are available within the article and its Supplementary Information file. Source data are provided with this paper.

## Source data

Source Data
