# Supplementary material for: Sirt6 attenuates chondrocyte senescence and osteoarthritis progression
Source: Nat Commun. 2022 Dec 10;13:7658. doi: 10.1038/s41467-022-35424-w (PMC9741608; doi:10.1038/s41467-022-35424-w)
Supplement: Supplementary file 1 — Supplementary Information [file 41467_2022_35424_MOESM1_ESM.pdf]

Sirt6 attenuates chondrocyte senescence and osteoarthritis progression

Ji et al.

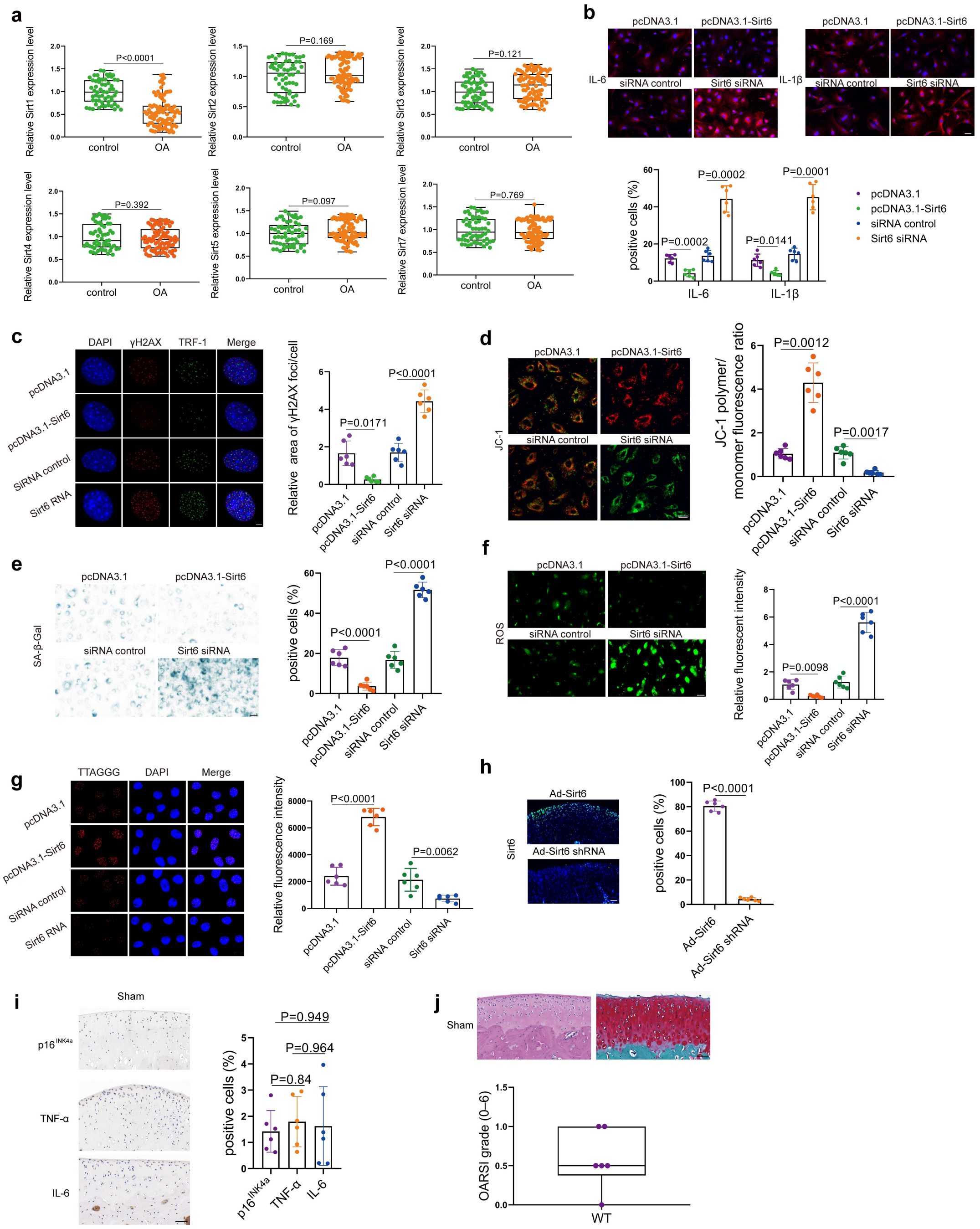

Supplementary Fig. 1 Chondrocyte senescence-associated secretory phenotype. a qPCR of other Sirtuins (Sirt1, 2, 3, 4, 5 and 7) levels in cartilage tissues of control subjects (n=60) and OA patients (n=90). **b** Representative immunofluorescent images showing the IL-6 and IL-1β expression levels in human OA chondrocytes that were

transfected by pcDNA3.1-Sirt6, Sirt6 siRNA or their corresponding controls for 48 hours. n=6 independent biological replicates per group. **c-f** Representative immunofluorescent images of DNA damage, mitochondrial membrane potential, SA-β-Gal positivity and ROS level in IL1β-induced SW1353 cells that were transfected by pcDNA3.1-Sirt6, Sirt6 siRNA or their corresponding controls for 48 hours. n=6 independent biological replicates per group. **g** Representative fluorescence microscopy images of telomere FISH analysis in human OA chondrocytes were transfected by pcDNA3.1-Sirt6, Sirt6 siRNA or their corresponding controls for 48 hours. n=6 independent biological replicates per group. **h** The efficiency the in vivo Sirt6 knockout and overexpression. n=6 mice per group. **i** Immunohistochemistry staining of $p16^{INK4a}$, TNF-α and IL-6 expression in articular cartilage of mice undergoing sham surgery. n=6 mice per group. **j** Safranin O/fast green (OARSI score) and H&E staining of knee joints of mice undergoing sham surgery. n=6 mice per group. Scar bar: **j** 50 μm, **b, d, e, h, i** 20 μm, **f, g** 10 μm, **c** 5 μm. Data are presented as the mean ± s.e.m (**b-i**) or boxplots (**a, j**). P values are from two-tailed Mann-Whitney *U* test (**a**), two-tailed unpaired t test with Welch's correction (**h**), one-way ANOVA test followed by Tukey's post hoc (**e, g, i**) or Brown-Forsythe and Welch ANOVA test followed by Tamhane's T2 post hoc analysis (**b, c, d, f**). Source data are provided as a Source Data file.

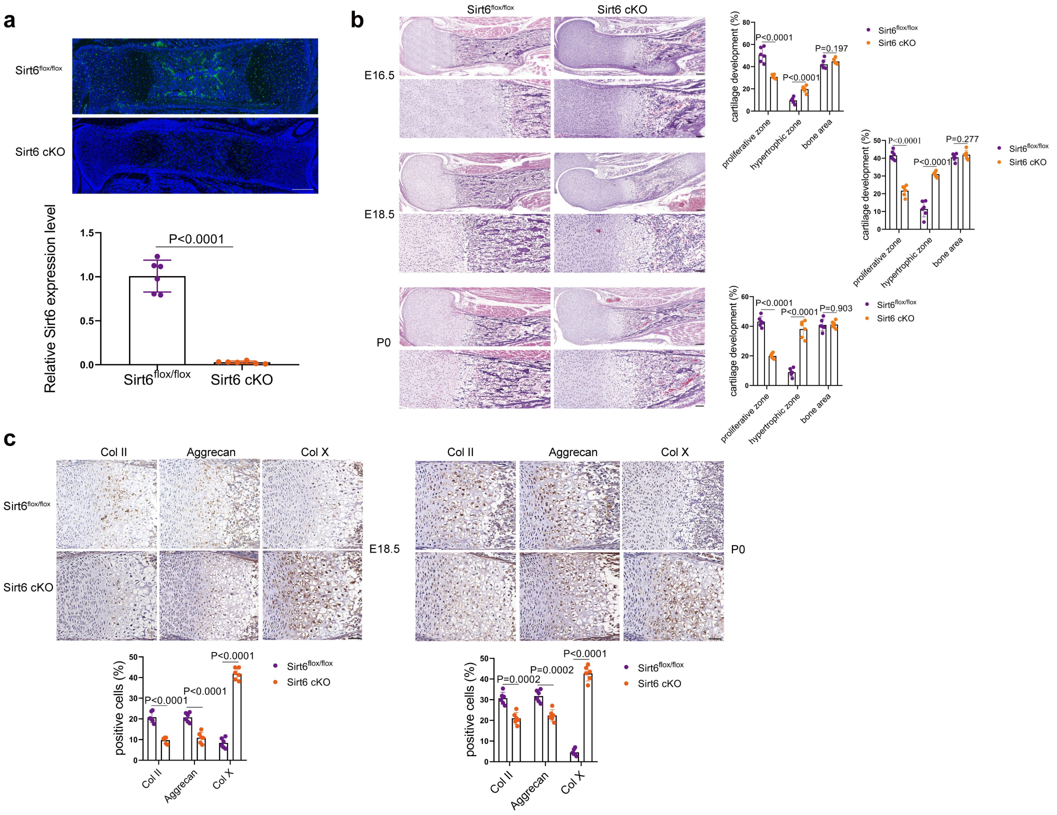

Supplementary Fig. 2 The pivotal role of Sirt6 in cartilage development. a Representative immunofluorescent images of tibial sections of mouse embryos (E16.5). RT-qPCR analysis of Sirt6 expression in Sirt6$^{flox/flox}$ and Sirt6 cKO mice. n=6 mice per group. **b** Masson trichrome staining of whole humerus of Sirt6$^{flox/flox}$ and Sirt6 cKO (E16.5, E18.5 and P0) and quantitative analysis of bone area, hypertophic zone and proliferative zone. n=6 mice per group. **c** Representative immunohistochemistry of Col II, Aggrecan and Col X in the tibia of Sirt6$^{flox/flox}$ and Sirt6 cKO mice at E18.5 and P0. n=6 mice per group. Scar bar: **a, b (upper)** 200 µm, **b (lower)** 50 µm, **c** 20 µm. Data are presented as the mean ± s.e.m. P values are from two-tailed unpaired t test (**b, c**) or two-tailed unpaired t test with Welch's correction (**a**). Source data are provided as a Source Data file.

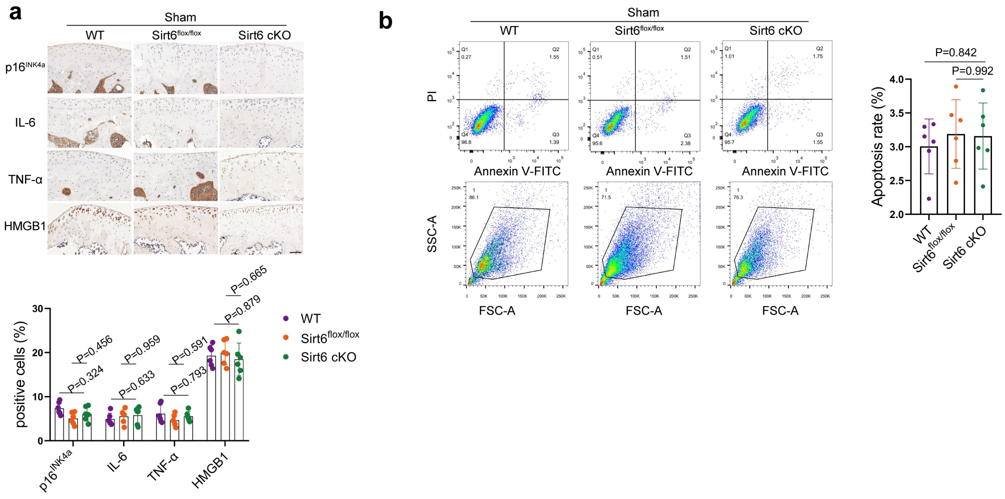

Supplementary Fig. 3 Expression pattern of senescence-associated secretory phenotype and chondrocyte viability. a Representative images of immunohistochemistry of p16^{INK4a}, IL-6, TNF-α and HMGB1 in cartilage tissues from the indicated groups (WT, Sirt6$^{flox/flox}$ and Sirt6 cKO mice subjected to sham surgery) at 8 weeks post surgery. n=6 mice per group. **b** Chondrocytes apoptosis was assayed by flow cytometry in the indicated groups (WT, Sirt6$^{flox/flox}$ and Sirt6 cKO mice subjected to sham surgery). n=6 mice per group. Scar bar: **a** 20 μm. Data are presented as the mean ± s.e.m. P values are from one-way ANOVA test followed by Tukey's post hoc (**a, b**). Source data are provided as a Source Data file.

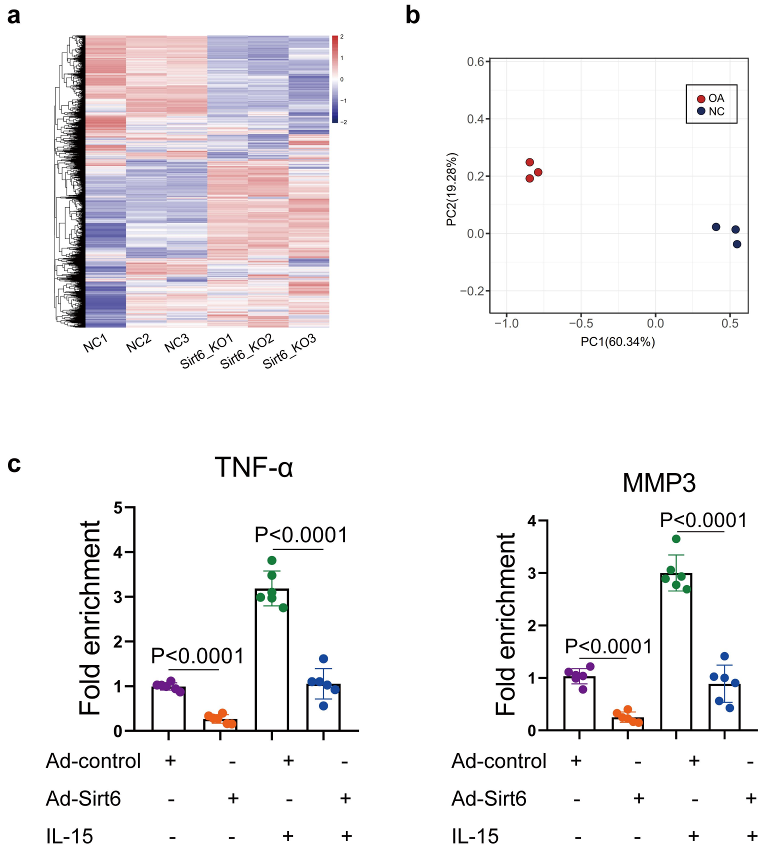

Supplementary Fig. 4 Involvement of IL-15/JAK3/STAT5 signaling pathway in chondrocyte senescence. a All genes expression profiles in Sirt6 KO chondrocytes vs controls. **b** Principal component analysis showing repeatability and consistency of samples. **c** ChIP assay analysis of human chondrocytes infected with Ad-control or Ad-Sirt6, then treated with or without IL-15. n=6 independent biological replicates per group. Data are presented as the mean ± s.e.m. P values are from Brown-Forsythe and Welch ANOVA test followed by Tamhane's T2 post hoc analysis (**c**). Source data are provided as a Source Data file.

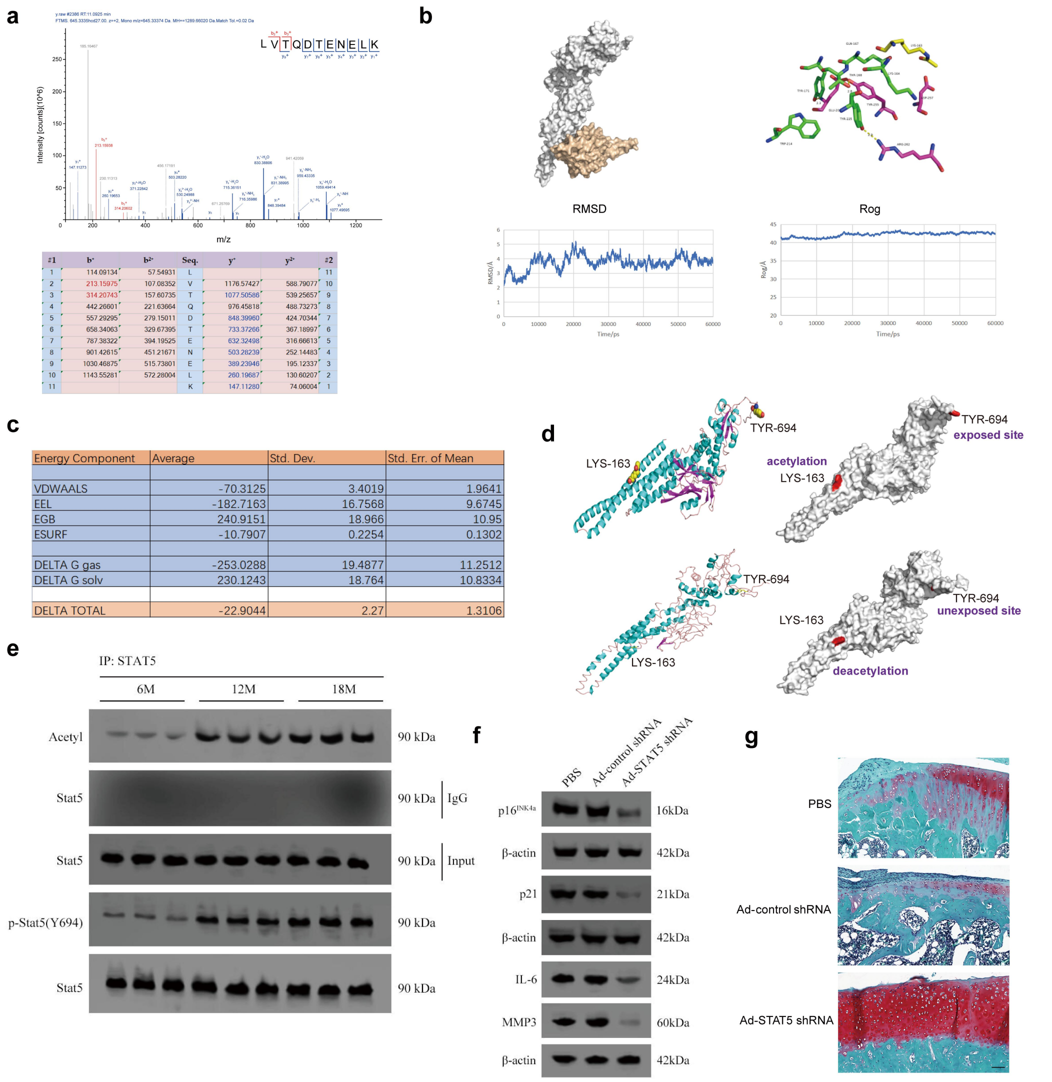

Supplementary Fig. 5 The interaction between Sirt6 and Stat5. a LC-MS/MS analysis showing deacetylation of key loci. **b** RMSD and radius of gyration (Rog) for Sirt6-Stat5 dynamics simulations. **c** The binding free energy between Sirt6 and Stat5. **d** The effect of acetylation of the lysine 163 (K163) of STAT5 on its Tyr 694 phosphorylation. **e** The western blot for acetylation and phosphorylation of STAT5 during chondrocyte senescence. n=3 independent biological replicates per group. **f** The influence of knockdown of STAT5 on SASP expressions. n=3 independent biological replicates per group. **g** The influence of knockdown of STAT5 on cartilage degradation. n=6 mice per group. Scale bar: **g** 50 μm.

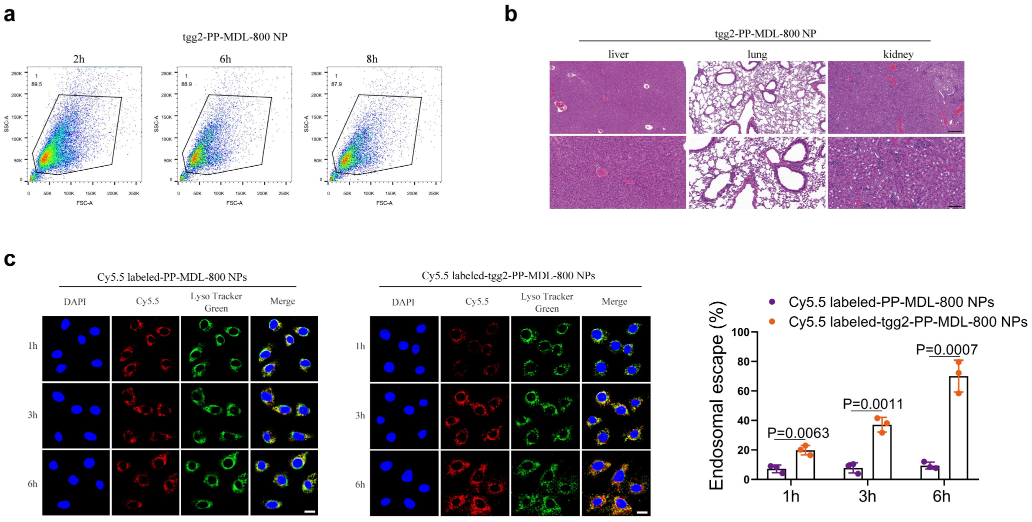

Supplementary Fig. 6 Evaluation of tgg2-PP-MDL-800 NP biological characteristics. a Gating strategies used for cell sorting. Gating strategy to sort apoptotic cells under tgg2-PP-MDL-800 NP treatment at 2, 6 and 8 hours. **b** H&E analysis for liver, kidney and lungs of mice at 3 months after IA injection of tgg2-PP-MDL-800 NP. n=6 mice per group. **c** Intracellular distribution of Cy5.5 labeled-PP-MDL-800 NPs and Cy5.5 labeled-tgg2-PP-MDL-800 NPs in SW1353 cells. n=3 independent biological replicates per group. Scale bar: **b (upper)** 100 µm, **b (lower)** 50 µm, **c** 20 µm. Data are presented as the mean ± s.e.m. P values are from two-tailed unpaired Student's t-test (**c**). Source data are provided as a Source Data file.

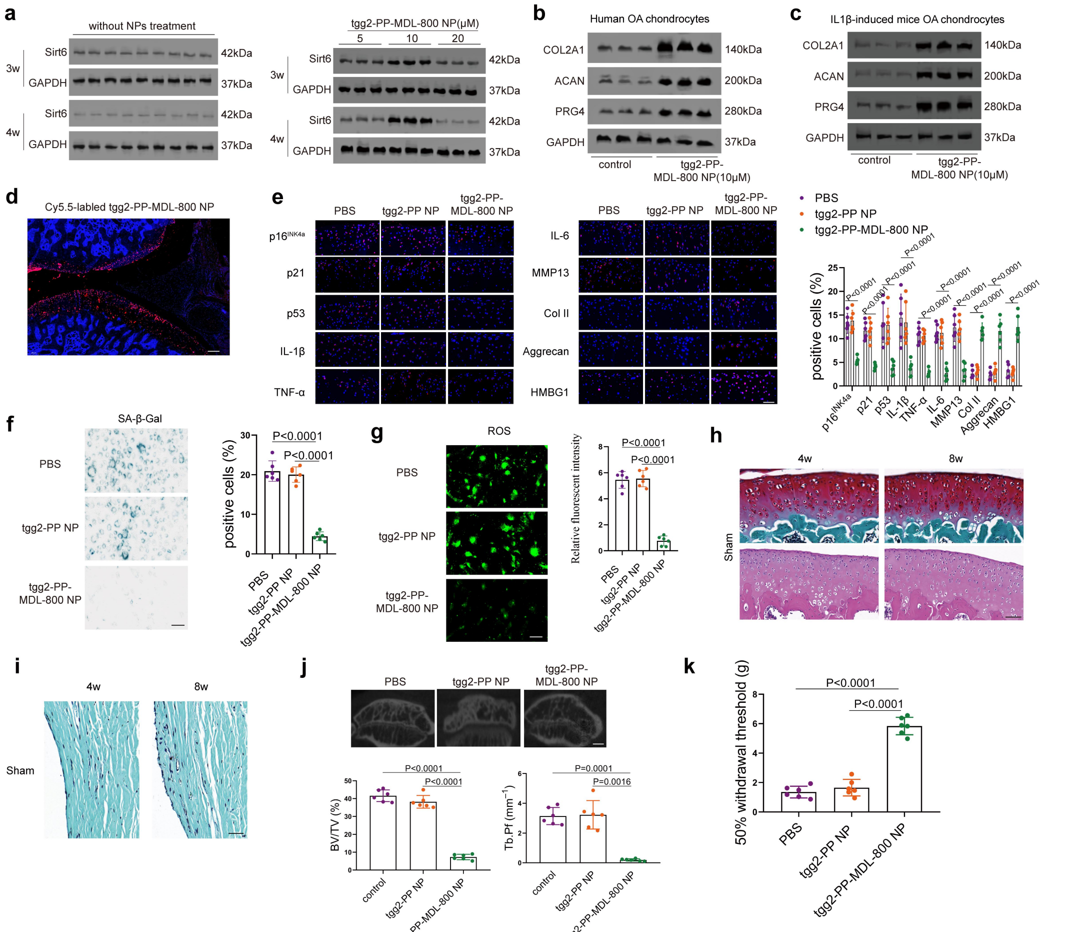

Supplementary Fig. 7 The therapeutic value of tgg2-PP-MDL-800 NP in OA. a The Sirt6 level in human OA chondrocytes without NPs treatment (left). The Sirt6 level in human OA chondrocytes treated with tgg2-PP-MDL-800 NP (5, 10 and 20μM) for 3 and 4 weeks (right). n=3 independent biological replicates per group. **b** The proteins levels of COL2A1, ACAN and PRG4 in human OA chondrocytes treated with control or tgg2-PP-MDL-800 NP (10μM) for 48 hours. n=3 independent biological replicates per group. **c** The proteins levels of COL2A1, ACAN and PRG4 in IL1β-induced mice OA chondrocytes treated with control or tgg2-PP-MDL-800 NP (10μM) for 48 hours. n=3 independent biological replicates per group. **d** The injected nanoparticles (Cy5.5 labeled-tgg2-PP-MDL-800 NP) distributed inside the joint. n=6 mice per group. **e** Representative immunofluorescent images of indicated markers in these cartilage tissues of OA mice model from different treatment groups. n=6

independent biological replicates per group. **f, g** SA-β-Gal positivity (f) and ROS level (g) was analyzed in chondrocytes of OA mice model treated by PBS, tgg2-PP NP or tgg2-PP-MDL-800 NP. n=6 independent biological replicates per group. **h, i** As controls, Safranin O-fast green staining of cartilage and synovial tissues from mice undergoing sham surgery at 4 and 8 weeks. n=6 mice per group. **j** Quantitative micro-CT analysis of tibial subchondral bone with trabecular bone volume per total volume (BV/TV) and trabecular bone pattern factor (Tb.Pf) in PBS, tgg2-PP NP or tgg2-PP-MDL-800 NP-treated DMM mice at 8 weeks. n=6 mice per group. **k** IA injection of tgg2-PP-MDL-800 NP reduced pain sensitivity induced by OA. The Von Frey test was performed in the 3-month-old mice receiving tgg2-PP-MDL-800 NP injection at the age of 12 weeks. n=6 mice per group. Scar bar: **j** 2 mm, **d, h, i** 50 μm, **f** 20 μm, **e, g** 10 μm. Data are presented as the mean ± s.e.m. P values are from one-way ANOVA test followed by Tukey's post hoc (**e, f, g, BV/TV in j, k**) or Brown-Forsythe and Welch ANOVA test followed by Tamhane's T2 post hoc analysis (**Tb.Pf in j**). Source data are provided as a Source Data file.

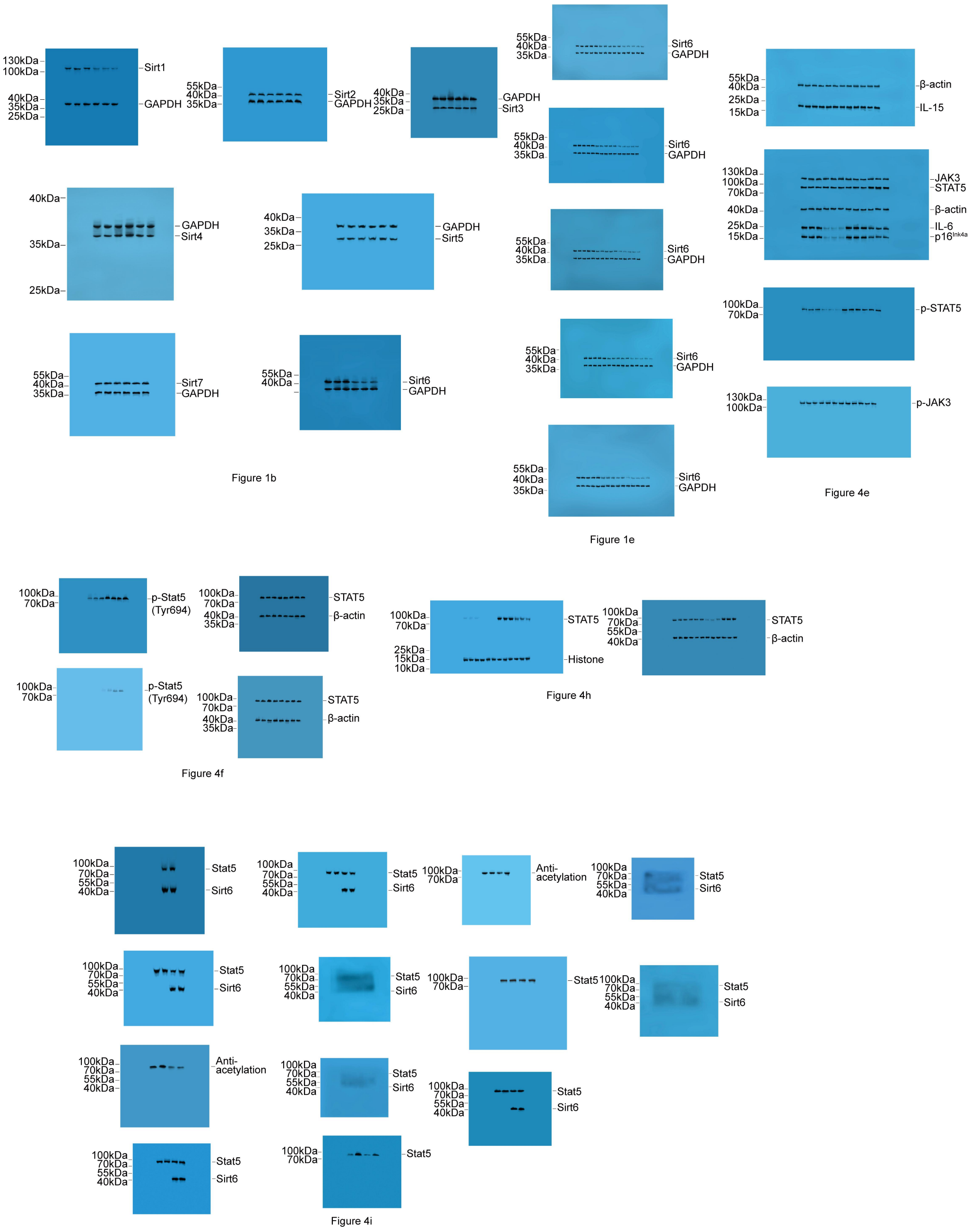

Supplementary Fig. 8 Full scans of important western blots (Fig. 1b, e and Fig. 4e, f, h, i).

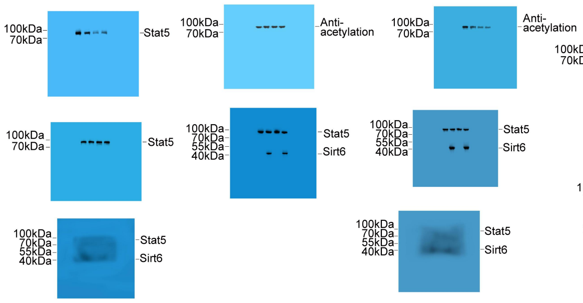

Figure 5e

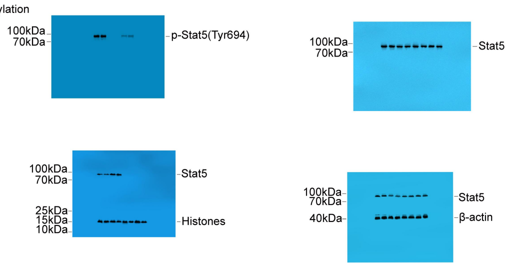

Figure 5f

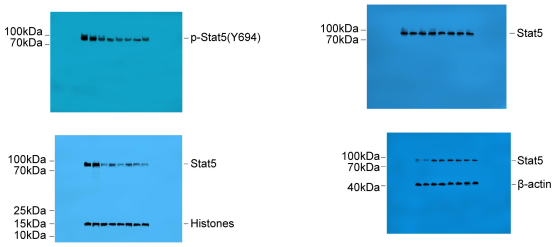

Figure 5i

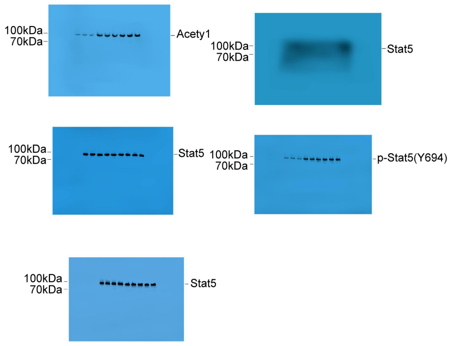

Supplementary Figure 5e

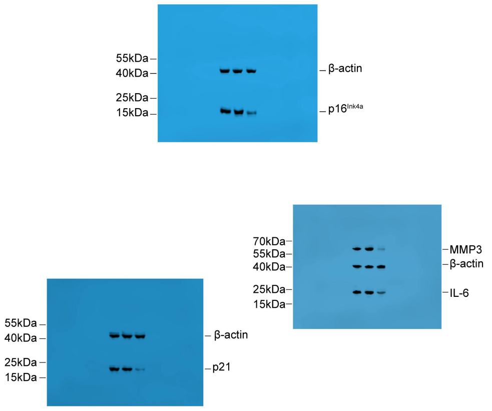

Supplementary Figure 5f

Supplementary Fig. 9 Full scans of important western blots (Fig. 5e, f, i and Supplementary Fig. 5e, f).

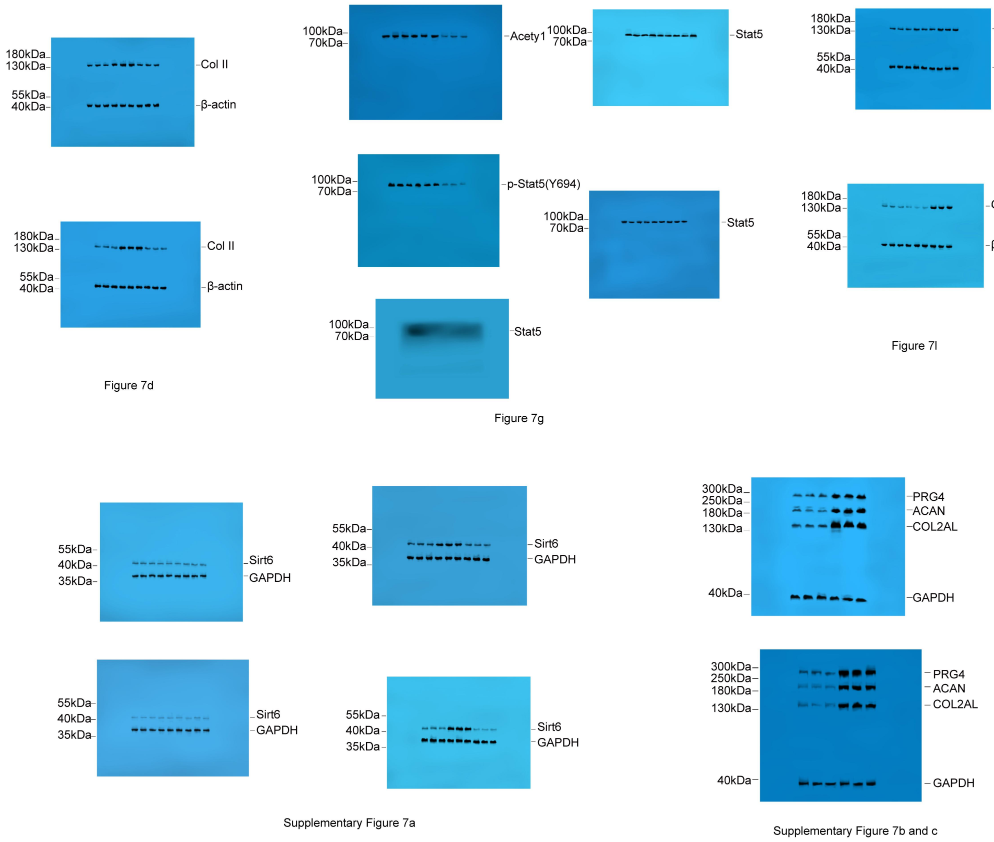

Supplementary Fig. 10 Full scans of important western blots (Fig. 7d, g, i and Supplementary Fig. 7a, b, c).
