# Supplementary material for: Sirt6 attenuates chondrocyte senescence and osteoarthritis progression
Source: Nat Commun. 2022 Dec 10;13:7658. doi: 10.1038/s41467-022-35424-w (PMC9741608; doi:10.1038/s41467-022-35424-w)
Supplement: Supplementary file 2 — Reporting Summary [file 41467_2022_35424_MOESM2_ESM.pdf]

nature portfolio

Corresponding author(s): Ming liang Ji; Jun Lu
Last updated by author(s): Nov 12, 2022

Reporting Summary

Nature Portfolio wishes to improve the reproducibility of the work that we publish. This form provides structure for consistency and transparency in reporting. For further information on Nature Portfolio policies, see our Editorial Policies and the Editorial Policy Checklist.

Statistics

For all statistical analyses, confirm that the following items are present in the figure legend, table legend, main text, or Methods section.

n/a	Confirmed	
☐	☒	The exact sample size (*n*) for each experimental group/condition, given as a discrete number and unit of measurement
☐	☒	A statement on whether measurements were taken from distinct samples or whether the same sample was measured repeatedly
☐	☒	The statistical test(s) used AND whether they are one- or two-sided<br>*Only common tests should be described solely by name; describe more complex techniques in the Methods section.*
☐	☒	A description of all covariates tested
☐	☒	A description of any assumptions or corrections, such as tests of normality and adjustment for multiple comparisons
☐	☒	A full description of the statistical parameters including central tendency (e.g. means) or other basic estimates (e.g. regression coefficient) AND variation (e.g. standard deviation) or associated estimates of uncertainty (e.g. confidence intervals)
☐	☒	For null hypothesis testing, the test statistic (e.g. *F*, *t*, *r*) with confidence intervals, effect sizes, degrees of freedom and *P* value noted<br>*Give P values as exact values whenever suitable.*
☒	☐	For Bayesian analysis, information on the choice of priors and Markov chain Monte Carlo settings
☒	☐	For hierarchical and complex designs, identification of the appropriate level for tests and full reporting of outcomes
☐	☒	Estimates of effect sizes (e.g. Cohen's *d*, Pearson's *r*), indicating how they were calculated

Our web collection on statistics for biologists contains articles on many of the points above.

Software and code

Policy information about availability of computer code

Data collection	RNA quantity and quality were determined using a nanodrop (Thermo Scientififc, Waltham, MA, USA) . Quantitative polymerase chain reaction (PCR) amplification was performed with an ABI QuantStudio 5 (Applied Biosystem, Foster City, CA). Library quality was evaluated on the Agilent Bioanalyzer 2100 system (Agilent Technologies, CA, USA). Final libraries were sequenced on Illumina NovaSeq 6000 platform by 150bp paired-end reads. LC-MS/MS analysis was performed on an EASY-nLC 1000 HPLC system (Thermo Scientific). For invivo scannings of animals, Skyscan 1176 micro-CT scanner (Skyscan, Aartselaar, Belgium) was used. Radiographs of mouse knee joints were obtained using the Faxitron MX20 X-ray system. Cellular uptake and intracellular distribution were examined using FluoView FV1000 confocal microscope (Olympus). Dynamic light scattering (DLS) and transmission electron microscopy were used to evaluate nanoparticles. The fluorescence (Cell immunofluorescence and immunofluorescence staining) was visualized under CarlZeiss LSM710 confocal microscope (CarlZeiss, Oberkochen, Germany). For immunoblotting, The bands were detected with iBright FL1000 (Thermo Scientififc). The histological images were acquired using DS-Ri2 camera (Nikon).

Data analysis	All statistical analyses were performed using GraphPad Prism 8 (GraphPad Software Inc., La Jolla, CA, USA) and R software (version 4.2.1). For RNA-seq, reads were preprocessed and aligned to the reference genome Mus musculus (GRCm38/mm10) using HISAT v2.1.0. The reference genomes were downloaded from the UCSC genome browser (http://genome.ucsc.edu). Transcript assembly and abundance estimation were performed using StringTie v1.3.4d. The aligned reads were assembled into known, novel, and alternative splicing transcripts and the relative abundance of each transcript was quantified in read counts using StringTie v1.3.4d. For statistical analysis, genes with a read count value of zero at least in one sample were excluded. Filtered data were transformed into log2(read count+1) values and subjected to RLE normalization. Statistical significance of the differential expression was determined using nbinomWaldTest of DESeq2. Gene ontology analysis of differentially expressed genes was performed using Enrichr (http://amp.pharm.mssm.edu/Enrichr). Top-ranked terms from WikiPathways, Kyoto Encyclopedia of Genes and Genomes (KEGG), BioCarta, Panther, and GO were selected by their Enrichr P values. For GSEA, gene lists of RNA sequencing data were ranked based on the fold change values (GSEA software v4.0.3).

For manuscripts utilizing custom algorithms or software that are central to the research but not yet described in published literature, software must be made available to editors and reviewers. We strongly encourage code deposition in a community repository (e.g. GitHub). See the Nature Portfolio guidelines for submitting code & software for further information.

Data

Policy information about availability of data

All manuscripts must include a data availability statement. This statement should provide the following information, where applicable:

- Accession codes, unique identifiers, or web links for publicly available datasets
- A description of any restrictions on data availability
- For clinical datasets or third party data, please ensure that the statement adheres to our policy

The original RNA-seq data generated in this study have been deposited in the GEO database under accession code GSE206513. Transcriptome data for OA condition during the study are available in the GEO database (http://www.ncbi.nlm.nih.gov/geo). All other relevant data supporting the findings of this study are available within the article and its Supplementary Information file. Source data are provided with this paper.

Human research participants

Policy information about studies involving human research participants and Sex and Gender in Research.

Reporting on sex and gender	Articular cartilage samples were sourced from 90 OA patients (female 83; male 7) who underwent total knee arthroplasty. As controls, 60 specimens (female 56; male 4) were obtained from individuals undergoing amputation surgery due to different pathological conditions.
Population characteristics	Articular cartilage samples were sourced from 90 OA patients (a median age of 61 years) who underwent total knee arthroplasty. As controls, 60 specimens (a median age of 60 years) were obtained from individuals undergoing amputation surgery due to different pathological conditions. The two groups were also matched for BMI. Upon separation of cartilage from bone tissue, the cartilage was immediately snap-frozen in liquid nitrogen.
Recruitment	Articular cartilage samples were sourced from 90 OA patients (female 83; male 7) who underwent total knee arthroplasty. As controls, 60 specimens (female 56; male 4) were obtained from individuals undergoing amputation surgery due to different pathological conditions. There was no self-selection bias in this study.
Ethics oversight	This study protocol was approved by the ethics committee of Zhongda hospital, and full written consents were obtained before the operative procedure.

Note that full information on the approval of the study protocol must also be provided in the manuscript.

Field-specific reporting

Please select the one below that is the best fit for your research. If you are not sure, read the appropriate sections before making your selection.

☒ Life sciences ☐ Behavioural & social sciences ☐ Ecological, evolutionary & environmental sciences

For a reference copy of the document with all sections, see nature.com/documents/nr-reporting-summary-flat.pdf

Life sciences study design

All studies must disclose on these points even when the disclosure is negative.

Sample size	Sample size was chosen to ensure an adequate statistical power.
Data exclusions	No data were excluded from our study.
Replication	Reproducibility of experimental findings was assessed by performing experiments with independent biological replicates separately collected. Experimental variation is reported in the applicable figures as standard error of the mean

Randomization	Cell cultures were randomly assigned to each experimental group. Animals were also randomly assigned to each experimental group, except in experiments that require specific genotypes.
Blinding	All samples were evaluated in a blinded manner. Comprehensive histological evaluation of cartilage tissues was conducted by two orthopedic pathologists. The observers were blinded to the genotype, feeding, or surgical condition of the mice.

Reporting for specific materials, systems and methods

We require information from authors about some types of materials, experimental systems and methods used in many studies. Here, indicate whether each material, system or method listed is relevant to your study. If you are not sure if a list item applies to your research, read the appropriate section before selecting a response.

Materials & experimental systems

n/a	Involved in the study
☐	☒ Antibodies
☐	☒ Eukaryotic cell lines
☒	☐ Palaeontology and archaeology
☐	☒ Animals and other organisms
☒	☐ Clinical data
☒	☐ Dual use research of concern

Methods

n/a	Involved in the study
☒	☐ ChIP-seq
☐	☒ Flow cytometry
☒	☐ MRI-based neuroimaging

Antibodies

Antibodies used

Immunoblotting
Sirt1 (1:1000, Cell Signaling Technology, #8469), Sirt2 (1:1000, Cell Signaling Technology, #12672), Sirt3 (1:1000, Cell Signaling Technology, #2627), Sirt4 (1:500, Santa Cruz Biotechnology, sc135797), Sirt5 (1:1000, Cell Signaling Technology, #8779), Sirt6 (1:1000, Cell Signaling Technology, #12486), Sirt7 (1:1000, Cell Signaling Technology, #5360), p16INK4a (1:1000; Abcam, ab270058), IL-6 (1:500, Abcam, ab6672), IL-15 (1:1000, Abcam, ab134177), JAK3 (1:2000, Abcam, ab45141), p-JAK3 (1:1000, Cell Signaling Technology, #5031), STAT5 (1:1000, Cell Signaling Technology, #25656), p-STAT5 (1:1000, Cell Signaling Technology, #4322), Col2A1 (1:500, Abcam, ab34712), ACAN (1:1000, Abcam, ab36861), PRG4 (1:1000; Abcam, ab28484), Histone (1:1500, Abcam, ab1791), GAPDH (1:2000, Abcam, ab8245) and beta-actin (1:1000, Cell Signaling Technology, 4967). The membranes were incubated with horseradish peroxidase (HRP)-linked anti-rabbit IgG (1:1000, Cell Signaling Technology, #7074) or HRP-linked anti-mouse IgG (1:1000, Cell Signaling Technology, #7076)
Cell immunofluorescence
p16INK4a (1:1000, Cell Signaling Technology, #18769), TNF-α (1:500; Abcam, ab1793), IL-6 (1:1000; Abcam, ab246703), IL-1β (1:100, Abcam, ab156791), P21 (1:800, Cell Signaling Technology, #2947), p53 (1:1000, Cell Signaling Technology, #2527), HMBG1 (1:2000; Abcam, ab18256), p-JAK3 (1:1000; Abcam, ab45141), STAT5 (1:200, Cell Signaling Technology, #25656), p-STAT5 (1:100, Cell Signaling Technology, #4322) and Alexa Fluor 555 (1:100, Abcam, ab150078)- or Alexa Fluor 488 (1:1000, Abcam, ab150077)-conjugated secondary antibodies
Immunohistochemistry
p16INK4a (1:1000; Abcam, ab241543), TNF-α (1:500; Abcam, ab220210), IL-6 (1:100; Abcam, ab290735), Col II (1:200; Abcam, ab34712), ACAN (1:500; Abcam, ab186414), Col X (1:1000; Abcam, ab49945) , p21 (1:50, Cell Signaling Technology, #2947), p53 (1:100, Cell Signaling Technology, #48818), IL-1β (1:200, Cell Signaling Technology, #12242) and HMBG1 (1:400; Abcam, ab79823)

Validation

Sirt1 (https://www.cellsignal.com/products/primary-antibodies/sirt1-1f3-mouse-mab/8469?site-search-type=Products&N=4294956287&Ntt=sirt1&fromPage=plp); Sirt2 (https://www.cellsignal.com/products/primary-antibodies/sirt2-d4s6j-rabbit-mab/12672?site-search-type=Products&N=4294956287&Ntt=sirt2&fromPage=plp), Sirt3 (https://www.cellsignal.com/products/primary-antibodies/sirt3-c73e3-rabbit-mab/2627?site-search-type=Products&N=4294956287&Ntt=sirt3&fromPage=plp), Sirt4 (https://www.scbt.com/zh/p/sirt4-antibody-95-1?requestFrom=search), Sirt5 (https://www.cellsignal.com/products/primary-antibodies/sirt5-d5e11-rabbit-mab/8779?site-search-type=Products&N=4294956287&Ntt=sirt5&fromPage=plp), Sirt6 (https://www.cellsignal.com/products/primary-antibodies/sirt6-d8d12-rabbit-mab/12486?site-search-type=Products&N=4294956287&Ntt=sirt6&fromPage=plp), Sirt7 (https://www.cellsignal.com/products/primary-antibodies/sirt7-d3k5a-rabbit-mab/5360?site-search-type=Products&N=4294956287&Ntt=sirt7&fromPage=plp), p16INK4a (https://www.abcam.cn/cdkn2ap16ink4a-antibody-epr24167-43-ab270058.html), IL-6 (https://www.abcam.cn/il-6-antibody-ab6672.html), IL-15 (https://www.abcam.cn/il-15-antibody-epr1542y-ab134177.html), JAK3 (https://www.abcam.cn/jak3-antibody-ep909y-ab45141.html), p-JAK3 (https://www.cellsignal.com/products/primary-antibodies/phospho-jak3-tyr980-981-d44e3-rabbit-mab/5031?site-search-type=Products&N=4294956287&Ntt=jak3&fromPage=plp), STAT5 (https://www.cellsignal.com/products/primary-antibodies/stat5-d3n2b-rabbit-mab/25656?site-search-type=Products&N=4294956287&Ntt=stat5&fromPage=plp), p-STAT5 (https://www.cellsignal.com/products/primary-antibodies/phospho-stat5-tyr694-d47e7-xp-rabbit-mab/4322?site-search-type=Products&N=4294956287&Ntt=p-stat5&fromPage=plp), Col2A1 (https://www.abcam.cn/collagen-ii-antibody-ab34712.html), ACAN (https://www.abcam.cn/aggrecan-antibody-ab36861.html), PRG4 (https://www.abcam.cn/lubricinmsf-antibody-ab28484.html), Histone (https://www.abcam.cn/histone-h3-antibody-nuclear-marker-and-chip-grade-ab1791.html), GAPDH (https://www.abcam.cn/gapdh-antibody-6c5-loading-control-ab8245.html), beta-actin (https://www.cellsignal.com/products/primary-antibodies/b-actin-antibody/4967), horseradish peroxidase (HRP)-linked anti-rabbit IgG (https://www.cellsignal.com/products/secondary-antibodies/anti-rabbit-igg-hrp-linked-antibody/7074?site-search-type=Products&N=4294956287&Ntt=%237074&fromPage=plp&_requestid=87469), HRP-linked anti-mouse IgG (https://www.cellsignal.com/products/secondary-antibodies/anti-mouse-igg-hrp-linked-antibody/7076).

Eukaryotic cell lines

Policy information about cell lines and Sex and Gender in Research

Cell line source(s)	SW1353 cells were purchased from ATCC (Cat No. HTB-94) and cultured according to the culture methods of ATCC.
Authentication	each cell line has been authenticated by American Type Culture Collection
Mycoplasma contamination	All cell lines tested negative for mycoplasma contamination
Commonly misidentified lines (See ICLAC register)	*Name any commonly misidentified cell lines used in the study and provide a rationale for their use.*

Animals and other research organisms

Policy information about studies involving animals; ARRIVE guidelines recommended for reporting animal research, and Sex and Gender in Research

Laboratory animals	Sirt6flox/flox and Col2a1-CreERT2 were purchased from Jackson Laboratories (Bar Harbor, ME, USA). Sirt6flox/flox and Sirt6 cKO (8 weeks old) mice were injected intraperitoneally with tamoxifen (100 μg/g body weight) daily for 5 days. Then, a surgically induced DMM OA model was performed on the knee joints of them (10-week-old). Animals were maintained under constant temperature (23-25°C), circulating air and humidity (45-65%) with a 12 h:12 h light/dark cycle. Mice had free access to food and water.
Wild animals	The study did not involve wild animals.
Reporting on sex	To generate male Col2a1-CreERT2;Sirt6flox/flox mice, male Sirt6flox/flox mice were mated with female Col2a1-CreERT2 mice to produce Col2a1-CreERT2; Sirt6flox/+ mice, which were then mated with Sirt6flox/flox mice. Male mice were used for DMM model and aging evaluation in this study. The number of mice used in this study was provided in the figure legends.
Field-collected samples	The study did not involve samples collected from the field.
Ethics oversight	The experimental protocol was approved by and performed in accordance with protocols from the Institutional Animal Care and Use Committee of southeast university.

Note that full information on the approval of the study protocol must also be provided in the manuscript.

Flow Cytometry

Plots

Confirm that:

☒ The axis labels state the marker and fluorochrome used (e.g. CD4-FITC).

☒ The axis scales are clearly visible. Include numbers along axes only for bottom left plot of group (a 'group' is an analysis of identical markers).

☒ All plots are contour plots with outliers or pseudocolor plots.

☒ A numerical value for number of cells or percentage (with statistics) is provided.

Methodology

Sample preparation	cultured primary mice chondrocytes
Instrument	EpicsAltra (Beckman Coulter, CA, USA) FCM
Software	flowjo7.6
Cell population abundance	Annexin V-FITC was employed to quantitatively determine the percentage of cells undergoing apoptosis. It relies on the property of cells to lose membrane asymmetry in the early phase of apoptosis. In apoptotic cells, the membrane phospholipid phosphatidylserine is translocated from the inner leaflet of the plasma membrane to the outer leaflet, thereby exposing phosphatidylserine to the external enviro
Gating strategy	Cells that were positively stained with Annexin V-FITC and negatively stained for PI were considered apoptosis. Cells that were positively stained for both Annexin V-FITC and PI were considered necrosis.

☒ Tick this box to confirm that a figure exemplifying the gating strategy is provided in the Supplementary Information.
